# Chemical Synthesis of Ubiquitin, Ubiquitin-Based Probes, and Diubiquitin**

**DOI:** 10.1002/anie.201005995

**Published:** 2010-11-29

**Authors:** Farid El Oualid, Remco Merkx, Reggy Ekkebus, Dharjath S Hameed, Judith J Smit, Annemieke de Jong, Henk Hilkmann, Titia K Sixma, Huib Ovaa

**Affiliations:** Division of Cell Biology, Netherlands Cancer InstitutePlesmanlaan 121, 1066 CX Amsterdam (The Netherlands) Fax: (+31) 20-512-2029 E-mail: h.ovaa@nki.nl, Homepage: http://research.nki.nl/Ovaalab/; Division of Biochemistry, Netherlands Cancer InstitutePlesmanlaan 121, 1066 CX Amsterdam (The Netherlands)

**Keywords:** chemical ligation, peptides, solid-phase synthesis, synthetic methods, ubiquitin

Post-translational modification of proteins with ubiquitin (Ub) and Ub chains controls protein breakdown by the proteasome, cellular localization of proteins, transcriptional activity, and DNA repair.[1] Ubiquitin is a highly conserved 76 amino acid protein that can be linked to target proteins through an isopeptide bond between the C-terminal carboxylate of Ub and the ɛ-amine of a lysine residue or N terminus of the target protein. Ubiquitin is able to form chains by self-conjugation onto any of its seven lysine residues (namely, K6, K11, K33, K27, K29, K48, and K63). Although all the linkages have been identified in cells,[2] only K48 and K63 linkages have been thoroughly studied so far. The conjugation of ubiquitin requires the concerted action of E1, E2, and E3 enzymes, defined combinations of which provide specificity for the protein target and the nature of the Ub chain topoisomers. The E1 enzyme initiates the cascade by activating Ub at the expense of ATP to form an E1-Ub thioester between the cysteine residue of the E1 active site and the C-terminal carboxylate of Ub. This E1-Ub thioester serves as a donor of activated Ub that then enters the complex enzymatic conjugation cascade.

The ability to generate Ub polymers biochemically is currently limited to the generation of K11, K48, and K63 topoisomers,[3] a strategy that requires prior identification and production of specific E2 enzymes.[4] Moreover, the generation of Ub mutants by biochemical methods is largely limited by the repertoire of natural amino acids. Therefore, reliable routes towards site-specifically labeled Ub derivatives, Ub-based reagents, and conjugates are needed to provide the scientific community with the research reagents they need.

Methods for the chemical synthesis of Ub have already been reported. Recent modular procedures based on the ligation of segments provide the best overall yields.[5a,b] However, the modular character introduces extensive purification procudures, thus making the procedures unsuitable for the automated parallel generation of reagents. Previously reported linear Fmoc-based (Fmoc=9-fluorenylmethoxycarbonyl) syntheses[5c–h] of Ub led to low yields (≤4 %) and modest purity at best. Several (semisynthetic) strategies towards Ub have been reported that allow the construction of isopeptide-linked Ub conjugates. The first approach[6] uses a photolabile auxiliary that assists native chemical ligation[7] of a recombinant Ub thioester. Other approaches rely on thiolysine-based chemical ligation handles,[8] and afford native isopeptide linkages after ligation to recombinant Ub thioester and subsequent desulfurization.[9] Thiolysine-mediated ligation was also used very recently in segment-based strategies to construct diubiquitins (diUb),[10ab] while K6- and K29-linked diUbs were obtained by a strategy that relies on a genetic code expanded to the incorporation of *N*^ɛ^-Boc-lysine (Boc=*tert*-butoxycarbonyl).[10c] In addition, various approaches towards isosteres of Ub isopeptide conjugates have been reported recently.[11]

Despite all the developments mentioned, more powerful and rapid approaches are still needed to obtain Ub derivatives and conjugates in sufficient quantities. Therefore, we have developed a high-yielding Fmoc-based linear solid-phase peptide synthesis (SPPS) of Ub that allows the incorporation of desired tags and mutations as well as specific C-terminal modification and the construction of any diUb conjugate in a straightforward manner. By using this method we have produced Ub, numerous Ub mutants, and conjugates on a 25 μmol scale with consistent purities and yields over 200 times.

**Figure 1:**
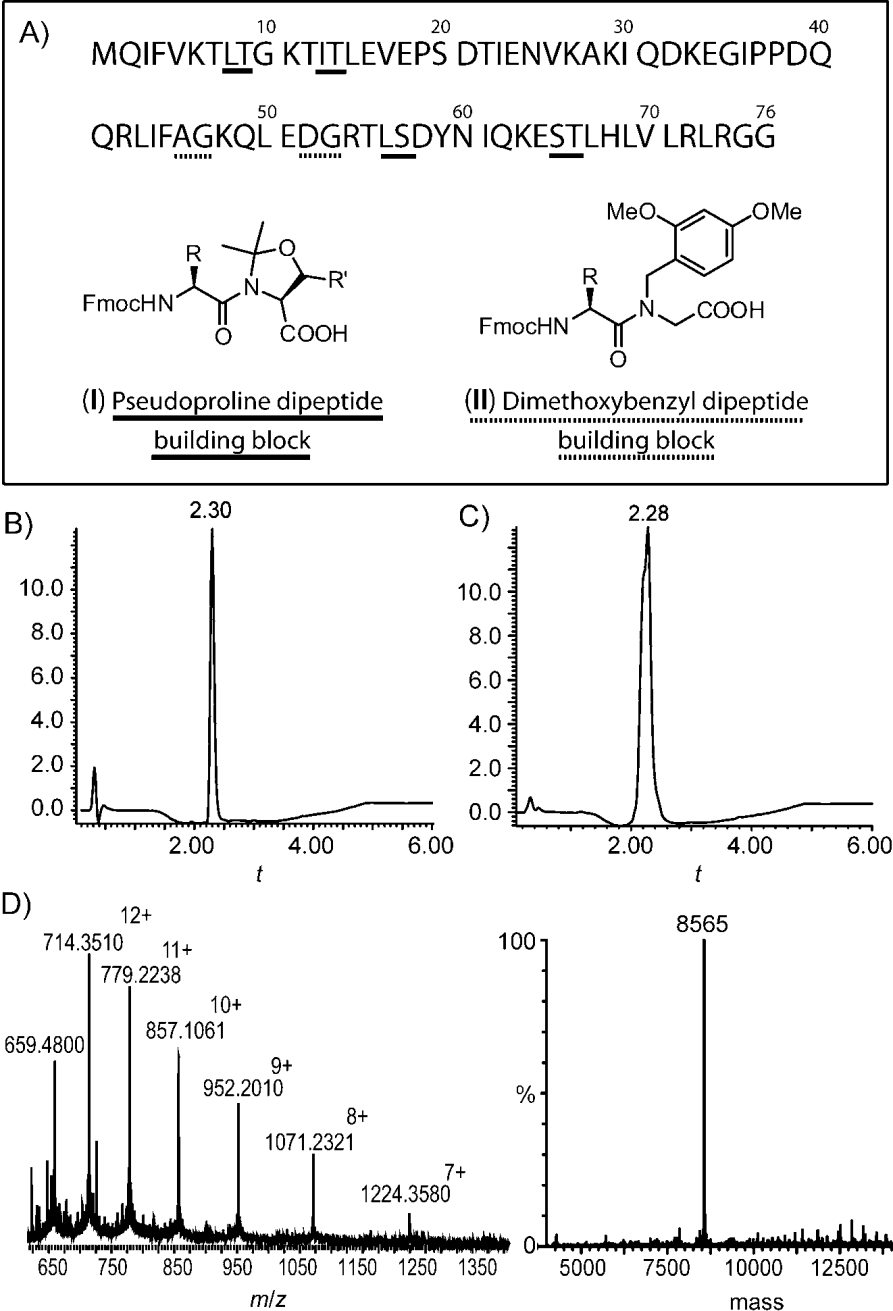
A) Amino acid sequence of Ub containing the set of dipeptide building blocks used in our Fmoc-SPPS of Ub; L8T9, I13T14, L56S57, and S65T66 were replaced by the corresponding pseudoproline dipeptide (**I**), A46G47 and D52G53 were replaced by their corresponding dimethoxybenzyl dipeptide (**II**). B) Liquid chromatography profile of commercial Ub and C) crude synthetic Ub. D) MS analysis of crude synthetic Ub, calcd 8565 Da, found: 8565 Da; the deconvoluted spectrum is shown on the right.

As linear syntheses yield the desired products directly and in parallel, which is a significant advantage over modular approaches, we revisited the linear chemical synthesis of Ub. We decided to investigate the incorporation of pseudoproline building blocks and dimethoxybenzyl (DMB) dipeptides (Figure 1 A), which prevent the formation of folded and/or aggregated intermediates on-resin, events that can hamper cleavage of the Fmoc group and/or further elongation of the Ub chain.[12]

We identified six positions in the Ub sequence where such dipeptide building blocks could be incorporated (Figure 1 A, see the Supporting Information for the selection of the building blocks). Using a Wang resin and standard coupling conditions (namely, 4 equiv Fmoc-protected amino acid, 4 equiv PyBOP, 8 equiv DIPEA, and single coupling reactions), simultaneous incorporation of all the six selected building blocks led to the synthesis of Ub in high yield (54 % yield of the crude product) and 14 % after refolding (see the Supporting Information) and purification by cation-exchange chromatography (Figure 1). A control experiment without these dipeptide building blocks did not give a defined product. Incorporation of the standard Fmoc amino acids at specific positions (S65T66 or L56S57) instead of the corresponding pseudoproline building blocks did afford Ub, but in very low yield. Omission of any of the other dipeptide building blocks led to an unproductive synthesis.

**Figure 2:**
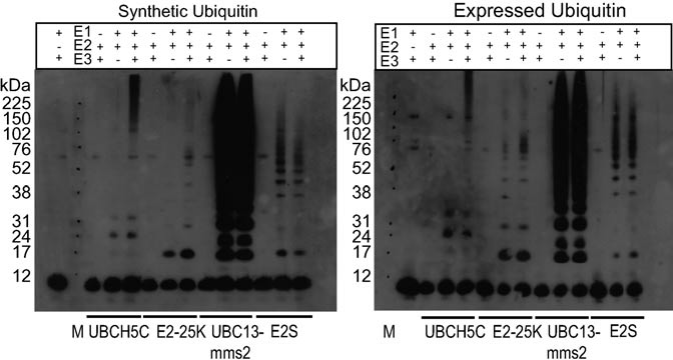
Comparison of the chain-forming capability of synthetic ubiquitin (left) with expressed ubiquitin (right) evaluated on an anti-Ub Western blot. The ability to form ubiquitin linkages through different lysine residues was compared in the presence of E1 and E2 as indicated, and with or without the E3 ligase Triad1. E1=Uba1 (500 nm), various E2s (2 μm); E3=Triad1 (1 μm), Ub (15 μm), ATP (3 mm), 30 °C, 2 1/2 h. In this assay all E2s promote chain formation of mixed chains (UbcH5c), K48 (E2-25K), K63 (Ubc13 mms2), or K11 linkages (E2S). The reaction with UbcH5c and E2-25K is stimulated by the presence of the E3 ligase Triad1. Synthetic and expressed ubiquitin form all ubiquitin chain types equally well.

Correct folding of the purified synthetic Ub was verified by circular dichroism (CD) spectroscopy (see the Supporting Information). To further verify the correct folding and thus biochemical function we compared synthetic and recombinant Ub in enzymatic ligation experiments (Figure 2). This proved that synthetic Ub is incorporated with the same efficiency as recombinant Ub into different chain topologies.

With a productive synthesis of Ub in hand, we synthesized various Ub fusions. His_6_- and HA epitope-tagged N-terminal Ub fusions could be generated efficiently, while site-selective N-terminal modification with various labels provided N-terminally labeled 5-carboxytetramethylrhodamine-Ub, 5(6)-carboxyfluorescein-Ub, and DOTA-Ub (DOTA=1,4,7,10-tetraazacyclododecane-1,4,7,10-tetraacetic acid) conjugates cleanly and in good yield upon purification (see the Supporting Information).

**Scheme 1:**
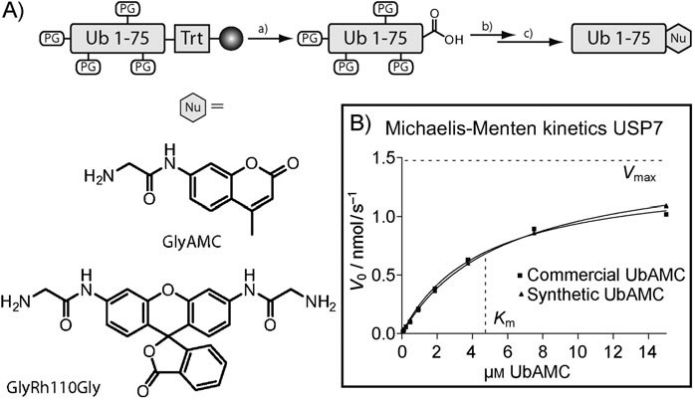
A) Synthesis of C-terminally modified Ub. PG=protecting group. a) HFIP/CH_2_Cl_2_, 30 min, RT; b) PyBOP, DIPEA, Nu, CH_2_Cl_2_, 16 h, RT; c) TFA/*i*Pr_3_SiH/H_2_O 3 h, RT. DIPEA=*N*,*N*-diisopropylethylamine, HFIP=1,1,1,3,3,3-hexafluoro-2-propanol, PyBOP=benzotriazol-1-yl-oxytripyrrolidinophosphonium hexafluorophosphate, TFA=trifluoroacetic acid. B) Turnover of commercial and synthetic UbAMC by USP7 shows identical Michaelis–Menten kinetics.

Having a general entry into Ub fusions and chemical mutants in hand we focused on generating C-terminal Ub fusions, since the combined set of chemical synthesis, ligation, and C-terminal modification provides entry into virtually any desired Ub derivative. For this purpose we synthesized full-length Ub on a hyper-acid-labile trityl resin (Scheme 1) with the N-terminal methionine residue protected with a Boc group. Total deprotection of this product with 95 % TFA gave Ub in the same purity and yield as the synthesis on Wang resin.

Protected synthetic Ub(1-75) with a free C-terminal carboxylate was then generated through selective cleavage from the resin with 20 % hexafluoro-2-propanol in CH_2_Cl_2_.[13] This product, which is fully soluble in neat dichloromethane was then condensed with GlyAMC and GlyRhodamine110Gly to yield UbAMC and UbRh110Gly in 6 % and 5 % overall yield, respectively, after deprotection and purification (Scheme 1). These Ub conjugates serve as fluorogenic substrates to measure the activity of deubiquitinating enzymes (DUBs).[14] Efficient turnover of synthetic UbAMC and UbRh110Gly was confirmed upon their incubation with the DUBs HAUSP/USP7 and UCH-L3 (see the Supporting Information). No significant difference between the turnover of the synthetic and commercial UbAMC by HAUSP/USP7 could be observed, and identical Michaelis–Menten constants were obtained (Scheme 1 B).

**Figure 3:**
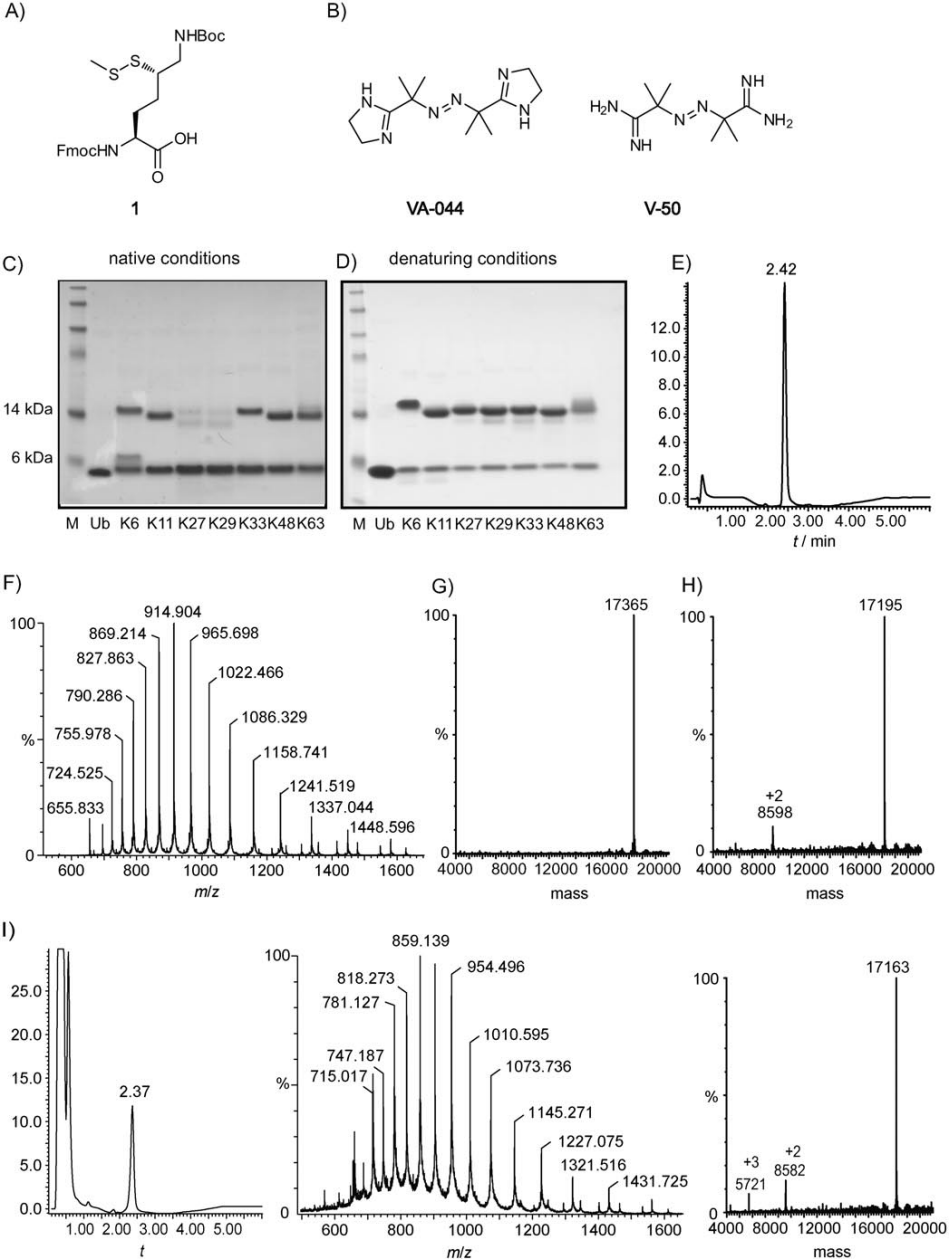
A) Structure of Fmoc- and methyldisulfide-protected δ-thiolysine building block **1**. B) Structure of radical initiators VA-044 and V-50. C) SDS-PAGE analysis of diUb ligation under native conditions by using in situ E1-generated Ub thioester: 50 mm sodium phosphate buffer pH 8, 6 mm ATP, 6 mm MgCl_2_, 50 μm Ub, 50 μm Ub K to δ-thiolysine/G76 V mutant, 250 nm E1, 50 mm MPAA. D) SDS-PAGE analysis of DiUb ligation under denaturing conditions: 6 m Gdn⋅HCl pH 8, 10 mg mL^−1^ UbMESNa, 10 mg mL^−1^ Ub K to δ-thiolysine/G76 V mutant, 100 mm MPAA, 50 mm TCEP. E) Liquid chromatography trace and F) mass spectrum of purified K33-linked diUb ligation product (δ-thiolysine intermediate). G) The deconvoluted mass spectrum of the ligation product showed a product with a mass that corresponds to the disulfide product of MPAA and δ-thiolysine of the diUb ligation product. H) Addition of 50 mm TCEP to the purified product gave the free thiol. I) Radical-mediated desulfurization of the K33-linked diUb ligation product resulted in clean formation of the native K33-linked diUb. Conditions: 6 m Gdn⋅HCl, 0.1 m sodium phosphate (pH 6.5) diUb conjugate (12 μm), 200 mm V-50, 250 mm TCEP, 40 mm glutathione, 60 °C, overnight incubation.

Next, we investigated whether we could construct higher order Ub conjugates. For this we chemically mutated individual lysine residues into δ-thiolysine residues. We incorporated the methyldisulfide-protected δ-thiolysine building block **1** (Figure 3 A; see the Supporting Information for synthetic details) into the Ub sequence to generate all seven individual lysine to δ-thiolysine methyldisulfide mutants, including a Gly76 to Val mutation to prevent processing by the E1 enzyme. After refolding and purification, mutants were obtained in 6–9 % overall yield (see the Supporting Information).

We investigated the ability of these double mutants to participate in the formation of diUb topoisomers. We first focussed on in situ E1-mediated thioester formation and concomitant native chemical ligation.[15] Initial reactions using 2-mercaptoethanesulfonic sodium salt (MESNa) as a thiol resulted in no detectable ligation. The use of the native chemical ligation catalyst 4-mercaptophenylpropionic acid (MPAA),[16] however, proved effective for the productive formation of diUb, as evidenced by analysis of the crude reaction mixture by sodium dodecasulfate polyacrylamide gel electrophoresis (SDS-PAGE; Figure 3 C). Production of K27 and K29 linkages proved difficult, most likely because residues K27 and K29 are the least accessible lysine residues in ubiquitin (see the Supporting Information). We therefore turned our attention to denaturing conditions to ligate purified ubiquitin thioester onto ubiquitin thiolysine mutants.

We first produced UbMESNa conveniently and in large quantities through E1-catalyzed transthioesterification of 100 μm Ub (50 mm sodium phosphate buffer pH 8, 500 nm E1, 10 mm adenosine triphosphate (ATP), 10 mm MgCl_2_, 100 mm MESNa, 5 h., 37 °C). Upon completion, the reaction mixture was acidified and purified, and consistently afforded ubiquitin thioester in over 80 % yield.

After optimization of the ligations under denaturing conditions, we identified the following general conditions as the most efficient for the generation of diUb topoisomers on a preparative scale: UbMESNa and Ub δ-thiolysine mutant are dissolved at 10 mg mL^−1^ in a 1:1 ratio in the ligation mixture (6 m guanidine hydrochloride (Gdn⋅HCl) pH 8, 50 mm tris(2-carboxyethyl)phosphine (TCEP) and 100 mm MPAA) and incubated overnight at 37 °C; next an additional amount of UbMESNa (0.5 equiv) is added to the ligation mixture to ensure full consumption of all the δ-thiolysine mutant. Gels of the crude ligation reactions are shown in Figure 3 D. After preparative HPLC, the anticipated δ-thiolysine-linked diUb conjugates were isolated as MPAA disulfides in all cases (Figure 3 D–G) on a multimilligram scale in yields ranging from 35 to 72 % (See the Supporting Information).

Attempts at radical-mediated desulfurizations at 37 °C by using the radical initiator VA-044 (Figure 3 B), glutathione (40 mm), and TCEP (250 mm) proved unsuccessful. Treatment of the K33-linked diUb conjugate (0.5 mg in 2.5 mL 6 m Gdn⋅HCl in 0.1 m sodium phosphate pH 6.5) with the alternative radical initiator V-50 (200 mm, Figure 3 B), 40 mm glutathione, and 250 mm TCEP at 60 °C led to a clean and complete desulfurization[9] after incubation overnight (Figure 3 I; see also the Supporting Information). This desulfurization procedure proved applicable on a preparative scale to all of the remaining linkages (see the Supporting Information).

In conclusion, we have shown that the Fmoc-SPPS of Ub reported here enables the parallel incorporation of desired tags, labels, and mutations in high yields with high purities. It also proved possible to introduce C-terminal modifications to Ub, thereby allowing the synthetic construction of various Ub-based reagents. This synthetic approach to specifically labeled derivatives offers significant advantages over the established intein method,[14c] which is largely limited to the natural amino acids. Finally, with our Ub Fmoc-SPPS methodology we have synthesized all seven possible δ-thiolysine-Ub mutants and used these for the construction of all diUb topoisomers in a native manner. Recently, three independent reports described routes to diUb conjugates.[10] The straightforward linear synthesis of Ub and Ub mutants that we describe here, combined with the efficient production of the UbMESNa thioester, now allows a more convenient preparation of diUb conjugates. Having routine strategies for the chemical construction of Ub mutants, Ub chains, or specific C-terminal modifications, virtually any Ub derivative is now within practical reach. We believe that the versatility of the methods reported here will accelerate the pace of research into the biology of Ub, thereby opening novel avenues for research and drug discovery.
