# Supplementary material for: Chemical Synthesis of Ubiquitin, Ubiquitin-Based Probes, and Diubiquitin
Source: Angew Chem Int Ed Engl. 2010 Nov 29;49(52):10149–53. doi: 10.1002/anie.201005995 (PMC3021723; doi:10.1002/anie.201005995)
Supplement: Supplementary file 1 [file anie0049-10149-SD1.pdf]

Angewandte Chemie
Eine Zeitschrift der Gesellschaft Deutscher Chemiker

Supporting Information

© Wiley-VCH 2010

69451 Weinheim, Germany

Chemical Synthesis of Ubiquitin, Ubiquitin-Based Probes, and Diubiquitin**

*Farid El Oualid, Remco Merkx, Reggy Ekkebus, Dharjath S. Hameed, Judith J. Smit, Annemieke de Jong, Henk Hilkmann, Titia K. Sixma, and Huib Ovaa**

anie_201005995_sm_miscellaneous_information.pdf

General. General reagents were obtained from Sigma Aldrich, Fluka and Acros and used as received. (5R)-5-hydroxy-L-lysine dihydrochloride monohydrate was purchased from Sigma Aldrich. Solvents were purchased from BIOSOLVE or Aldrich and, where necessary, dried over molecular sieves (4Å for DCM, DMF and 3Å for MeOH). Peptide synthesis reagents were purchased from Novabiochem. Analytical thin layer chromatography was performed on aluminium sheets precoated with silica gel 60 F^{254} using 20% ninhydrin in ethanol and heating by a heatgun. Column chromatography was carried out on silica gel (0.035-0.070 mm, 90Å, Acros). Nuclear magnetic resonance spectra (^{1}H-NMR, ^{13}C-NMR and COSY) were determined in MeOD-d_4 (^{1}H δ 4.87 ppm; ^{13}C δ 49.15 ppm) using a Bruker ARX 400 Spectrometer (^{1}H: 400 MHz, ^{13}C: 100 MHz) at 298 K, unless indicated otherwise. Peak shapes in NMR spectra are indicated with the symbols 'd' (doublet), 'dd' (double doublet), 's' (singlet) triplet and 'm' (multiplet). Chemical shifts (δ) are given in ppm and coupling constants *J* in Hz. LC-MS measurements were performed on a system equipped with a Waters 2795 Seperation Module (Alliance HT), Waters 2996 Photodiode Array Detector (190-750nm), Waters Alltima C18 (2.1x100mm, 3 μm), Waters Symmetry300^{TM} C4 (2.1x100mm, 3.5 μm) or Phenomenex Kinetex C18 (2.1x50, 2.6 μm) and LCT^{TM} Orthogonal Acceleration Time of Flight Mass Spectrometer. Samples were run using 2 mobile phases: A = 1% CH_3CN, 0.1% formic acid in water and B = 1% water and 0.1% formic acid in CH_3CN. Data processing was performed using Waters MassLynx Mass Spectrometry Software 4.1 (deconvulation with Maxent1 function).

LC-MS programs

Program 1: Waters AtlantisT3^{TM} C18, 2.1×100 mm, 3 μM); flow rate= 0.4 mL/min, runtime= 10 min, column T= 40°C. Gradient: 0 − 2 min: 5% B; 2 − 5 min: ⇨ 95% B; 5 − 7 min: 95% B.

Program 2: Waters Symmetry300^{TM} C4, 2.1×100 mm, 3.5 μM; flow rate = 0.2 mL/min, runtime= 30 min, column T= 40°C. Gradient: 0 - 2 min: ⇨ 5%B; 2 - 3 min: ⇨ 10% B; 3 – 17 min: ⇨ 90% B; 17 - 30 min: ⇨ 95% B.

Program 3: Phenomenex Kinetex C18, (2.1×50 mm), 2.6 μM); flow rate= 0.8 mL/min, runtime= 6 min, column T= 40°C. Gradient: 0 − 0.5 min: 5% B; 0.5 − 4 min: ⇨ 95% B; 4 − 5.5 min: 95% B.

Fmoc SPPS Strategy

SPPS was performed on a Syro II MultiSyntech Automated Peptide synthesizer using standard 9-fluorenylmethoxycarbonyl (Fmoc) based solid phase peptide chemistry at 25 μmol scale, using fourfold excess of amino acids relative to pre-loaded Fmoc amino acid Wang type resin (0.2 mmol/g, Applied Biosystems®) or pre-loaded Fmoc amino acid trityl resin (0.2 mmol/g, Rapp Polymere GmbH). The following protected amino acid,

pseudoproline and DMB dipeptide building blocks were uses during ubiquitin synthesis: Fmoc-L-Ala-OH, Fmoc-L-Arg-(Pbf)-OH, Fmoc-L-Asn(Trt)-OH, Fmoc-L-Asp(O*t*Bu)-OH, Fmoc-L-Gln(Trt)-OH, Fmoc-L-Glu(OtBu)-OH, Fmoc-L-Gly-OH, Fmoc-L-His(Trt)-OH, Fmoc-L-Ile-OH, Fmoc-L-Leu-OH, Fmoc-L-Lys(Boc)-OH, Fmoc-L-Met-OH; Fmoc-L-Phe-OH; Fmoc-L-Pro-OH; Fmoc-L-Ser(*t*Bu)-OH; Fmoc-L-Thr(*t*Bu)-OH, Fmoc-L-Tyr(*t*Bu)-OH, Fmoc-L-Val-OH, Fmoc-L-Ser(*t*Bu)-L-Thr($\Psi^{Me,Me}$pro)-OH, Fmoc-L-Leu-L-Ser($\Psi^{Me,Me}$pro)-OH, Fmoc-L-Ile-L-Thr($\Psi^{Me,Me}$pro)-OH; Fmoc-L-Leu-L-Thr($\Psi^{Me,Me}$pro)-OH, Fmoc-L-Asp(O*t*Bu)-(Dmb)Gly-OH and Fmoc-L-Ala-(Dmb)Gly-OH. All amino acid and dipeptide building blocks were dried overnight under high vacuum prior to use.

For the first 30 cycles:

- single couplings in NMP for 45 min using PyBOP (4 equiv) and DiPEA (8 equiv)
- Fmoc removal with 20% piperidine in NMP for 2×2 and 1×5 min.
- capping with a mixture of Ac_2O/DiPEA/HOBt in NMP at 500 mM, 125 mM and 15 mM respectively (3×1.2 mL, 2×2 and 1×5 min). This solution was prepared fresh on ice every 2 days.

After the first 30 cycles:

- the coupling time was extended to 60 minutes
- Fmoc deprotection with 20% piperidine in NMP for 4x3 minutes.
- in the case of Pro37 and Thr12 we observed that a single coupling onto this position can result in incomplete incorporation of these amino acids; this can be prevented by using double couplings (2×90 min) for these two residues.
- capping as described above.

Work-up

The resin was washed with diethylether and dried under high vacuum. Next, the polypeptide sequence was detached from the resin and deprotected by treatment with TFA/H_2O/Phenol/iPr_3SiH 90.5/5/2.5/2 v/v/v/v for 3 h. After washing the resin with 3×1 mL TFA/H_2O/Phenol/iPr_3SiH (90.5/5/2.5/2 v/v/v/v), the crude protein was precipitated with cold Et_2O/*n*-pentane 3:1 v/v. The precipitated protein was washed 3x with Et_2O, the pellet was dissolved in a mixture of H_2O/CH_3CN/HOAc (65/25/10 v/v/v) and finally lyophilized.

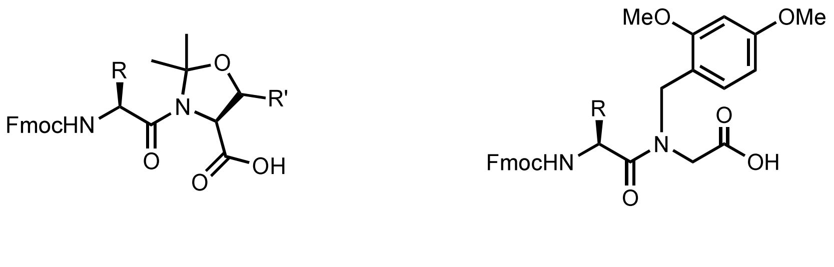

Figure S1A. General structure of special dipeptide building blocks used in our SPPS of the ubiquitin polypeptide sequence. Left: pseudoproline dipeptides, right: dimethoxybenzyl (DMB) dipeptides.

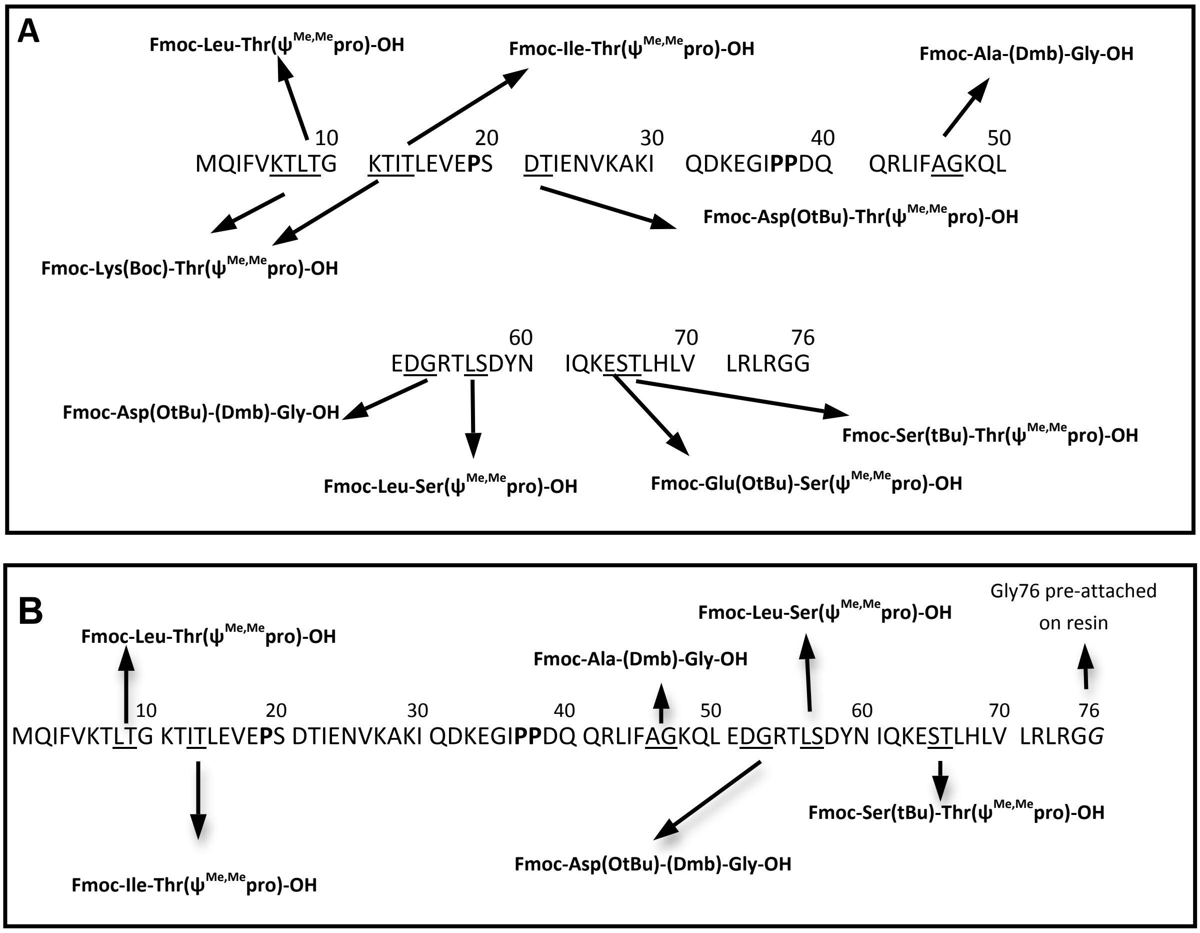

Figure S1B. (A) Analysis of ubiquitin peptide sequence for potential positions of commercially available special SPPS building blocks. Proline residues are highlighted in bold. (B) Final set of dipeptide building blocks used in our SPPS strategy of the ubiquitin polypeptide sequence.

From the peptide sequence of Ub, several positions can be identified that are suitable for incorporation of the pseudoproline- and dimethoxybenzyl dipeptide building blocks (Figure S1A). A schematic representation of this analysis is shown in Figure 1B. When incorporating these type of special SPPS dipeptides, several aspects should be taken into account as much as possible:[1]

- optimum separation between a special dipeptide and Pro is ≥ 5 amino acids
- optimum separation between two special dipeptides is ≥ 5 amino acids
- minimum separation is 2 amino acids
- if possible, insert dipeptide before a region of hydrophobic residues
- insertion very near the N- or C-terminus is not necessary since aggregation is not a problem here

Because the lysine residues in ubiquitin will be mutated, we chose to not incorparate any dipeptide building blocks at Lys6Thr7 and/or Lys11Thr12.

[1] NovaBiochem® catalog, synthesis notes.

Folding and purification of synthetic ubiquitin (mutants)

The crude Ub (mutant) is folded by taking it first up in a minimal amount of warm DMSO and then diluting the DMSO solution with 50 mM NaOAc pH 4.5 – the final DMSO concentration is kept as low as possible (2 – 10%). Next, the folded peptide is purified by cation chromatography using a MonoS column and a 0 ⇨ 1 M NaCl gradient in 50 mM NaOAc pH 4.5. Pure fractions are analysed by LC-MS and the 50 mM NaOAc pH 4.5 buffer containing ±0.18 M NaCl is exchanged for milliQ over a 3 kDa cutoff spin-column (Amicon® Ultra). The Ub (mutant) in milliQ is then lyophilized.

His_6-Ub: White powder (12.9 mg, 6%); LC-MS (program 3): R_t 2.18 min; MS ES+ (amu) calculated: 9388.19 $[M+H]^+$; found 9388 $[M+H]^+$.

HA-Ub: White powder (9.4 mg, 4%); LC-MS (program 3): R_t 2.38 min; MS ES+ (amu) calculated: 9649.5 $[M+H]^+$; found 9649 $[M+H]^+$.

UbK6δ-thiolysineG76V mutant: White powder (15.4 mg, 7.1%).
UbK11δ-thiolysineG76V mutant: White powder (16.2 mg, 7.5%).
UbK27δ-thiolysineG76V mutant: White powder (14.5 mg, 6.7%).
UbK29δ-thiolysineG76V mutant: White powder (19.1 mg, 8.8%).
UbK33δ-thiolysineG76V mutant: White powder (16.8 mg, 7.7%).
UbK48δ-thiolysineG76V mutant: White powder (18.6 mg, 8.6%).
UbK63δ-thiolysineG76V mutant: White powder (9.6 mg, 6.4%).

For LC-MS data of UbK➔δ-thiolysineG76V and UbK➔δ-thiolysine mutants, see LC-MS data section

General method for the N-terminal modification of Ub

The Ub(1-76) peptide sequence with a free N-terminus was synthesized on a Wang resin following the general procedure. For the modification reaction, a solution of the label (10 equiv), DIC (10 equiv) and HOBt (10 equiv) in NMP (800 μL) was incubated for 5 min and added to the resin-bound peptide (1 equiv). The mixture was gently shaken for 3h at room temperature before the resin was filtered and washed with NMP, DCM and Et_2O. Post-modification work-up including cleavage/deprotection, lyophilization and purification by cation chromatography were performed according to the general procedure.

CF-Ub

The modification was carried out following the general procedure using resin-bound Ub(1-76) (12.5 μmol), 5(6)-carboxyfluorescein (47.0 mg, 125 μmol), DIC (19.4 μL, 125 μmol) and HOBt (16.9 mg, 125 μmol), in DMF (800 μL). The product (10.1 mg, 9%) was obtained as a bright yellow solid. LC-MS (program 3): R_t 2.40 min; MS ES+ (amu) calculated: 8923.7 $[M+H]^+$; found 8924 $[M+H]^+$.

TAMRA-Ub

The modification was carried out following the general procedure using resin-bound Ub(1-76) (12.5 μmol), TAMRA (53.8 mg, 125 μmol), DIC (19.4 μL, 125 μmol) and HOBt (16.9 mg, 125 μmol), in DMF (800 μL). The product (15.2 mg, 14%) was obtained as a deep purple solid. LC-MS (program 3): R_t 2.35 min; MS ES+ (amu) calculated: 8977.8 $[M+H]^+$; found 8978 $[M+H]^+$.

DOTA-Ub

The modification was carried out following the general procedure using resin-bound Ub(1-76) (12.5 μmol), DOTA-tris-tert-butyl ester (71.6 mg, 125 μmol), DIC (19.4 μL, 125 μmol) and HOBt (16.9 mg, 125 μmol), in DMF (800 μL). The product (10.5 mg, 9%) was obtained as a white solid. LC-MS (program 3): R_t 2.28 min; MS ES+ (amu) calculated: 8951.8 $[M+H]^+$; found 8951 $[M+H]^+$.

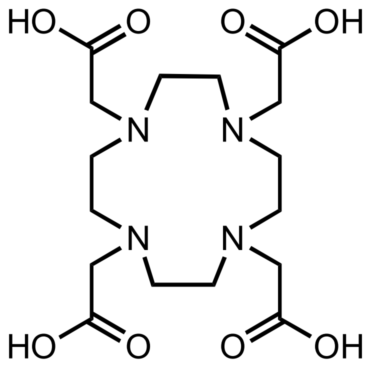

Figure S2. DOTA = 1,4,7,10-tetraazacyclododecane-N,N',N'',N'''-tetraacetic acid

Synthesis δ-thiolysine building block 4.[2]

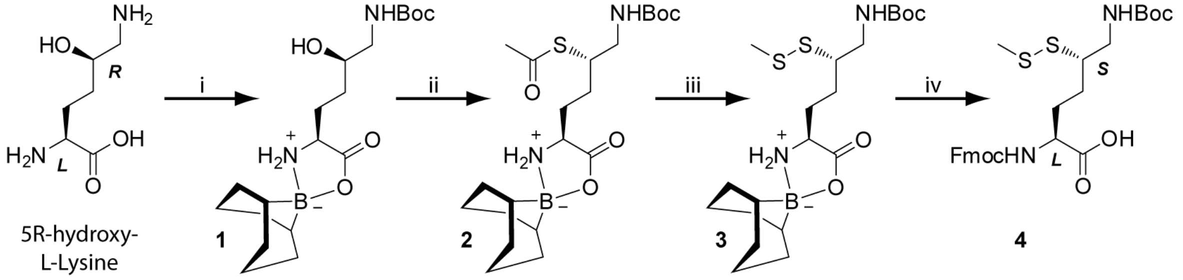

Scheme S1. Reagents and conditions: i) 1) 9-BBN, MeOH, 70°C, 4h, 2) Boc_2O, $NaHCO_3$, dioxane/H_2O, 0°C→RT, overnight, 89%, 2 steps; ii) 1) MsCl, Et_3N, CH_2Cl_2, 0°C→RT, 1 h, 2) KSAc, DMF, 65°C, 2½ h, 80%, 2 steps; iii) 1) NaOH, MeOH, 0°C→RT, 15 min., 2) Et_3N, CH_2Cl_2, MMTS, 1h, >99%, 2 steps; iv) 1) ethylenediamine, THF, 70°C, 2) FmocOSu, $NaHCO_3$, acetone/H_2O, 0°C→RT, overnight, 78%, 2 steps. 9-BBN= 9-borabicyclo[3.3.1]nonane, Boc= *tert*-butoxycarbonyl, DMF= *N,N*-dimethylformamide, MMTS= S-methyl methanethiosulfonate, MsCl= methanesulfonyl chloride, FmocOSu=N-(9-fluorenylmethoxy carbonyloxy) succinimide, THF= tetrahydrofuran.

N^{ε}*-tert*-Butoxycarbonyl-5*R*-hydroxy-L-lysinato-bicyclononylboron (1).

A 28% ammonia solution (aq. 10 mL) was added to (5R)-5-hydroxy-L-lysine dihydrochloride monohydrate (1.04 g, 4.11 mmol) at 0°C. After stirring for 30 min the solution was concentrated and the crystalline solid was dried in high vacuum before further use. The solid was added in one portion to a stirred solution of 9-BBN (1.2 g, 4.7 mmol) in hot methanol (20 mL). The reaction mixture was refluxed (ca. 3 h) under N_2 until a clear solution was obtained. After evaporation of the solvent, the residue was dissolved in 1,4-dioxane/water (2/3 v/v, 30 mL), cooled in an ice-bath and treated with $NaHCO_3$ (0.5 g) and Boc_2O (1.1 g). After stirring overnight, the reaction mixture was concentrated, diluted with brine and extracted with EtOAc. After drying ($MgSO_4$) and concentration, the product was purified over silica gel (n-hexane→EtOAc). Compound **1** (R_f = 0.4, EtOAc) was obtained as a white foam. Yield: 1.39 g, 3.63 mmol, 89% over 2 steps. *On a 45.7 mmol scale, the product was obtained (silica gel chromatography DCM → 10% MeOH/DCM) in an overall yield of 82%.* ^{1}H-NMR (400 MHz, MeOD-d4) δ 3.72 – 3.59 (m, 2H, H-α and H-δ), 3.12 – 3.01 (m, 2H, H-ε), 2.11 (m, 1H, H-β), 1.90 – 1.43 (m, CH-boron, H-β' and H-γ), 1.43 (s, 9H, tBu Boc), 0.57 (d, 2H, CH_2 boron, J = 16 Hz). ^{13}C-NMR (100 MHz, MeOD-d_4) δ 177.4 (C=O), 158.9 (C=O, Boc), 80.4 (C_q *t*Bu), 71.6 (CH), 56.5 (CH), 47.5 (CH_2), 32.7 – 32.4, 31.7 (CH_2), 28.9 (tBu, Boc), 28.4 (CH_2), 25.8 – 25.4 (CH_2). LC-MS (program 1): R_t = 6.9 min, MS ES+ (amu): 383.26 $[M+H]^+$, 765.01 $[M\text{-}M+H]^+$.

[2] Syed *et al.*, *Tetrahedron* **2004**, *60*, 5571 – 5575.

***N^ε-tert*-Butoxycarbonyl-5*S*-(*S*-acetyl)-L-lysinato-bicyclononylboron** (**2**). To a solution of **1** (1.35 g, 2.48 mmol) and Et_3N (730 µL, 5.24 mmol) at 0°C in dichloromethane (15 mL) was added MsCl (326 µL, 4.19 mmol). The reaction mixture was stirred for 1 hour when TLC analysis showed completion. The crude product was purified over silica gel (n-hexane/EtOAc 1:1→1:3) affording the mesylate (R_f = 0.8, EtOAc) as a foam. Yield: >99%. ^{1}H-NMR (400 MHz, MeOD-*d*$_4$) δ 6.40 (m, 1H), 5.83 (m, 1H), 4.98 (m, 1H), 4.69 (m, 1H), 3.70 (m, 2H, H-α and H-δ), 3.25 – 3.12 (m, partially obscured by MeOD-*d*$_4$ peak), 2.69 (s, 3H), 2.11 (m, 1H, H-β), 2.12 – 1.30 (m, CH-boron, H-β and H-γ), 1.20 (s, 9H, *t*Bu Boc), 0.57 (broad s, 2H, CH_2 boron). LC-MS (program 1): R_t = 7.3 min, MS ES+ (amu): 461.19 $[M+H]^+$, 920.77 $[M\text{-}M+H]^+$. Potassium acetate (1.75 eq, 10.9 mmol, 1.25 g) was added to a solution of the mesylate (1.0 eq, 2.87 g, 6.23 mmol) in dry DMF (58 mL). The reaction was stirred at 65°C for 3 hrs when TLC and LC-MS analysis showed completion. The DMF was evaporated and the concentrate was dissolved in EtOAc, washed with water and brine, dried, and concentrated. Yield after silica gel chromatography: 2.21 g, 5.05 mmol, 81%.^{1}H-NMR (400 MHz, MeOD-*d*$_4$) δ 3.64 (app dd, 2H, H-α and H-δ, J = 4.8 and 8.5 Hz), 3.55 (dd, 1H, H-ε, J = 5.8 and 14.2 Hz), 3.12 (dd, 1H, H-ε', J = 7.3 and 14.1 Hz), 2.33 (s, 3H), 2.10 – 1.30 (m, CH-boron, H-β and H-γ), 1.43 (s, 9H, *t*Bu Boc), 0.57 (broad s, 2H, CH_2 boron). ^{13}C-NMR (100 MHz, MeOD-*d*$_4$) δ 197.0 (C=O SAc), 177.1 (C=O), 158.8 (C=O, Boc), 80.5 (C_q *t*Bu), 55.9 (CH), 45.7 (CH_3), 45.1 (CH_2), 32.8, 32.7, 32.4 (4×CH_2), 30.9 (CH), 29.6, 29.3 (2×CH_2), 28.9 (*t*Bu, Boc), 28.4 (CH_2), 25.8, 25.4 (2×CH_2). LC-MS (program 1): R_t = 7.4 min, MS ES+ (amu): 441.28 $[M+H]^+$, 880.97 $[M\text{-}M+H]^+$.

***N^ε-tert*-Butoxycarbonyl-5*S*-(methyldisulfanyl)-L-lysinato-bicyclononylboron (3).** Thioacetate **2** (1.13 g, 2.5 mmol) was dissolved in methanol (15 mL) and treated with 1N NaOH solution (3 mL) for 15 min at 0°C. The reaction mixture was carefully neutralized with equimolar amounts of HOAc and concentrated. The concentrate was dissolved in ethyl acetate and washed with water and brine, dried ($MgSO_4$), and concentrated affording the crude thiol as an oil. ^{1}H-NMR (400 MHz, MeOD-*d*$_4$) δ 3.64 (app t, 1H, H-α J = 7.5 and 5.4 Hz), 3.28 (dd, 1H, H-ε, J = 7.6 and 14.1 Hz), 3.13 (dd, 1H, H-ε', J = 6.8 and 13.9 Hz), 2.87 (broad s, 1H, H-δ), 2.10 – 1.30 (m, CH-boron, H-β and H-γ), 1.43 (s, 9H, *t*Bu Boc), 0.57 (broad d, 2H, CH_2

boron, J = 13.9 Hz). ^{13}C-NMR (100 MHz, MeOD-d_4) δ 177.3 (C=O), ~159 (C=O, Boc, low intensity peak), 80.5 (C_q *t*Bu), 56.2 (CH), 49.1 (CH_2, partially obscured by MeOD-d_4), 41.5 (CH), 33.1, 32.8, 32.7, 32.5, 32.4, 29.7 (5×CH_2), 29.0 (CH), 28.9 (*t*Bu, Boc), 25.8, 25.4 (2×CH_2). Next, a degassed solution of the thiol in DCM (7 mL) was added dropwise (in 45 min, with a syringe pump) to a degassed solution of *S*-Methyl methanethiosulfonate (3 equiv, 6.9 mmol, 0.66 mL) and Et_3N (9 equiv, 2.76 mL, 20.4 mmol) in DCM (7 mL). The reaction mixture was stirred for 1 h when TLC analysis (n-hexane/EtOAc 1:3 v/v) showed completion. After evaporation of the DCM, the crude product was purified over silica gel (n-hexane/EtOAc 2:3 v/v) affording **3** (R_f = 0.8, EtOAc) as an oil. Yield: 1.1 g, 2.5 mmol, >99% over 2 steps. ^{1}H-NMR (400 MHz, MeOD-d_4) δ 3.68 (dd, 1H, H-α, J = 5.4 and 6.8 Hz), 3.29 (m, 2H, H-ε, obscured by MeOD-d_4), 2.89 (m, 1H, H-δ), 2.43 (s, 3H, SMe), 1.90 – 1.43 (m, CH-boron, H-β and H-γ), 1.45 (s, 9H, *t*Bu Boc), 0.58 (broad d, 2H, CH_2 boron, J = 12.3 Hz). ^{13}C-NMR (100 MHz, MeOD-d_4) δ 177.1 (C=O), 158.7 (C=O, Boc), 80.5 (C_q *t*Bu), 56.1, 52.7, 48.9 (3×CH), 45.0 (CH_2), 32.8, 32.7, 32.4, 29.6, 29.1 (6×CH_2), 28.9 (*t*Bu, Boc), 25.8, 25.4 (2×CH_2), 24.5, 18.5 (2×CH). LC-MS (program 1): R_t = 11.6 min, MS ES+ (amu): 445.18 $[M+H]^+$, 889.35 $[M\text{-}M+H]^+$.

***N*$^{\alpha}$-(Fluoren-9-ylmethoxycarbonyl)-*N*$^{\varepsilon}$-*tert*-Butoxycarbonyl-5*S*-(methyldisulfanyl-L-lysine (4).** Compound **3** (2.24 g, 5.0 mmol) was dissolved in THF (40 mL) and ethylene diamine (1.4 mL) was added. When the solution was heated (oil-bath ~70°C or heatgun) a white solid precipitated (9-BBN·ethylene diamine complex). The reaction mixture was cooled and the precipitate filtered over Hyflo®. The filtrate was concentrated and in case of more precipitate being formed, filtered again. Flash column chromatography (DCM → 40% MeOH in DCM, R_f = 0.4) gave *N*$^{\varepsilon}$*-tert-butoxycarbonyl-5S-(methyldisulfanyl)-L-lysine* as a gummy solid (1.27 g, 3.91 mmol, 78%). LC-MS (program 1): R_f = 5.7 min, ES+ (amu): 325.40 $[M+H]^+$, 649.39 $[M\text{-}M+H]^+$. Next, a solution of Fmoc-OSu (1.25 eq, 1.65 g, 4.881 mmol) in acetone (25 mL) was added to a cooled solution of *N*$^{\varepsilon}$*-tert-Butoxycarbonyl-5S-(methyldisulfanyl)-L-lysine* (1.27 g, 3.91 mmol) and $NaHCO_3$ (360 mg, 4.30 mmol, 1.1 eq) in acetone/H_2O (225 mL/50 mL). The reaction mixture was stirred overnight, analysed by TLC/LCMS, concentrated, acidified with 1N aq. $KHSO_4$ and extracted with EtOAc. The organic layer was dried ($MgSO_4$) and concentrated. Silica gel chromatography

(0 → 10% MeOH in DCM) gave **4** as an oil which formed a foam under high vacuum. Yield: 2.1 g, 3.9 mmol, 99%. ^{1}H-NMR (400 MHz, MeOD-d_4) δ 7.75 (d, 2H, Fmoc, J = 7.5 Hz), 7.64 (t, 2H, Fmoc, J = 6.5 Hz Hz), 7.37 (t, 2H, Fmoc, J = 7.3 Hz), 7.29 (double t, 2H, Fmoc, J = 1.2, 7.4 and 7.5 Hz), 4.31 (d, 2H, J = 6.6 Hz), 4.18 (t, 1H, J= 6.9 Hz), 4.12 (dd, 1H, 4.8 and 8.2 Hz), 3.18 (m, 1H), 2.79 (m, 1H), 2.35 (s, SMe), 2.15 (m, 1H), 1.92 (m, 1H), 1.73 (m, 1H), 1.59 (m, 1H), 1.40, 1.38 (s, *t*Bu Boc). ^{13}C-NMR (100 MHz, MeOD-d_4) δ 176.9 (C=O), 158.7 (C=O, Boc), 145.5, 145.3, 142.7 (C_q Fmoc), 128.9, 128.3, 126.4, 121.0 (CH Fmoc), 80.4 (C_q *t*Bu), 68.1 (CH_2), 55.7, 52.4 (2×CH), 48.5 (CH), 45.5 (CH_2), 30.6, 28.8 (2×CH_2), 28.9 (*t*Bu, Boc), 24.5 (CH). LC-MS (program 1): R_t = 7.5 min, MS ES+ (amu): 547.18 $[M+H]^+$.

General protocol for the expression and purification of Ubiquitin activating enzyme E1 (human, his tagged)

Uba1 was produced with a *C*-terminal his-tag in E. coli BL21(DE3) cells from a pET3a vector. Cells were induced by autoinduction and cultivated overnight at 15°C. The cells were lysed by a high-pressure EmulsiFlex-C5 device (Avestin, Mannheim, Germany) and cleared lysates were incubated with Talon beads. His-tagged Uba1 was eluted in 30 mM Tris/HCl pH 8.0, 100 mM NaCl, 5 mM β-mercaptoethanol and 500 mM Imidazole. The protein was further purified by anion exchange chromatography on a PorosQ column, eluting Uba1 with a linear NaCl gradient to 600 mM NaCl. Final purification was achieved by size exclusion chromatography (Superdex 200) in Tris/HCl pH8.0, 100 mM NaCl, 5 mM βME. The protein was concentrated by using an Ultracel YM30 filter (Centriprep, Millipore). The final yield was 14.5 mg (2.4 mg/mL).

General method for the C-terminal modification of Ub

The Ub(1-75) peptide sequence was synthesized on a trityl resin following the general procedure except for the final methionine residue () which was introduced as the corresponding Boc derivative. The resin bound polypeptide was treated with 5 mL of DCM/HFIP (4:1 v/v) for 30 min and filtered. The resin was rinsed with DCM (3×5 mL) and the combined filtrates were concentrated. The partially protected peptide residue (1 equiv) was redissolved in DCM and reacted with PyBOP (5 equiv) and an excess of the nucleophile and TEA. The reaction mixture was stirred over night at room temperature. The solvent was removed *in vacuo* and the residue

was treated with TFA/H_2O/TiS (95:2.5:2.5 v/v/v) for 3 h followed by precipitation with cold Et_2O/pentane 3:1 v/v. Further workup (*i.e.* lyophilization and purification by cation chromatography) was performed according to the general procedure.

UbAMC

The modification was carried out following the general procedure using resin-bound Ub(1-75) (25 µmol), 2-amino-N-(4-methyl-2-oxo-2H-chromen-7-yl)acetamide (H-Gly-AMC, 58 mg, 250 µmol),[3] PyBOP (65 mg, 125 µmol) and TEA (70 µL, 500 µmol), in DCM (5 mL). The product (13.8 mg, 6%) was obtained as a white solid. LC-MS (program 3): R_t 2.28 min; MS ES+ (amu) calculated: 8722.6 $[M+H]^+$; found 8722 $[M+H]^+$.

Ub-Rh110-Gly

The modification was carried out following the general procedure using resin-bound Ub(1-75) (25 µmol), glycine-rhodamine110-glycine (112 mg, 250 µmol),[4] PyBOP (65 mg, 125 µmol) and TEA (70 µL, 500 µmol), in DCM (5 mL). The product (10.47 mg, 5%) was obtained as a white solid. LC-MS (program 3): R_t 2.27 min; MS ES+ (amu) calculated: 8934.8 $[M+H]^+$; found 8935 $[M+H]^+$.

Determination of concentrations of ubiquitins for biochemical assays

Ubiquitins were dissolved in buffer and concentrations were determined by a Pierce 660 nM assay and a Ubiquitin standard curve.

Ub mutant	Measured concentration
Fluorescein-Ub	0.325 mg/ml
Ub-AMC	0.563 mg/ml
Ub-Rh110-Gly	0.975 mg/ml
TAMRA-Ub	0.438 mg/ml

Ligase assay - Ubiquitin chain formation

E1, E2s and Triad1 E3 ligase were produced as described (Marteijn *et al*, 2009). Ubiquitin chain formation was assayed using 15 µM ubiquitin, 0.5 µM human Uba1 as E1, 2 µM E2 as mentioned, in the presence and absence of 1 µM Triad1 as E3-ligase. Reactions were performed in 20 mM Hepes pH 7.5, 150 mM NaCl, 3 mM ATP, 2 µM $ZnCl_2$, 10 mM $MgCl_2$, 2 mM DTT, for 2.5 hours at 30°C and separated on a 4 – 12% NuPage gel in MES-buffer.

---

[3] Onitsuka *et al.*, *Biosci. Biotechnol. Biochem.* **2006**, *70*, 2836 – 2843.
[4] DiGlyRh110 can be purchased from Promega.

Ubiquitin-AMC assays

DUB activity on commercial (Sigma) and synthetic ubiquitin with a *C*-terminal fluorescent group, 7-amino-4-methylcoumarin (UbAMC) were performed at 25°C in buffer containing 50 mM Hepes pH 7.5, 100 mM NaCl, 1 mM EDTA, 0.05% (w⁄v) Tween20, 10 mM DTT. The concentrations Ubiquitin-AMC and DUB are specified in the figures. Assays were performed in "Non binding surface flat bottom low flange" black 384-well plates (Corning) in 30 µl reactions. Kinetic data was collected in intervals of 5 min using a Fluostar Optima fluorescence plate reader (BMG Labtechnologies) at excitation and emission wavelengths of 355 nm and 460 nm, respectively for UbAMC. Experimental data was processed using Prism 4.03 (GraphPad Software, Inc.).

Deubiquitinating enzymes UCH-L3 and USP7

UCH-L3 in pRSET vector was obtained from Dr. Keith Wilkinson. UCH-L3 was expressed in Escherichia coli and purified as described in: Larsen *et al. Biochemistry* **1996**, *35*, 6735–6744.

Usp7(206-1102) was expressed in E.coli from a synthetic construct and purified as described: (a) Shanmugham *et al. J. Am. Chem. Soc.* **2010**, *132*, 8834–8835; (b) Fernandez-Montalvan *et al. Febs J.* **2007**, *274*, 4256–4270.

UbAMC/ Ub-Rh110-Gly DUB assay

Assay buffer: 50 mM HEPES pH 7.5, 100 mM NaCl, 1 mM EDTA, 0.05% Tween20, 10 mM DTT

- Reactions (30 µL) were incubated for 30 min at 25°C, measured every 5 min.
- Proteins used: UbAMC (Commercial), UbAMC (Synthetic), Ub-Rh110-Gly (Synthetic), USP7 full length, UCH-L3

All assays contained 1nM DUB and the following concentrations of Ub:

	**1**	**2**	**3**	**4**	**5**	**6**	**7**	**8**	**9**
**Ub**	15 µM	7.5 µM	3.75 µM	1.88 µM	0.9 µM	0.45 µM	0.23 µM	0.12 µM	0.06 µM

Figure 3A. Hydrolysis of fluorogenic Ub derivatives by the deubiquitinating enzymes HAUSP/USP7 and UCH-L3. **A,B)** Commercial and synthetic UbAMC are treated side by side with HAUSP/USP7 and compared. **C)** Synthetic UbAMC was treated with UCH-L3. It was found that the synthetic UbAMC was hydrolyzed and thus recognized as a substrate by the DUB. In this case we did not include AMC to determine the maximum emission, therefore, V_{max} was not calculated. **D,E)** Ub-Rh110-Gly was treated with HAUSP/USP7 and UCH-L3. The synthetic Ub-Rh110-Gly was hydrolyzed and thus recognized as substrate by both DUBs. In this case we did not include Rh110-Gly to determine the maximum emission, therefore, V_{max} was not calculated. All assays contained 1 nM of enzyme, substrate concentration was varied.

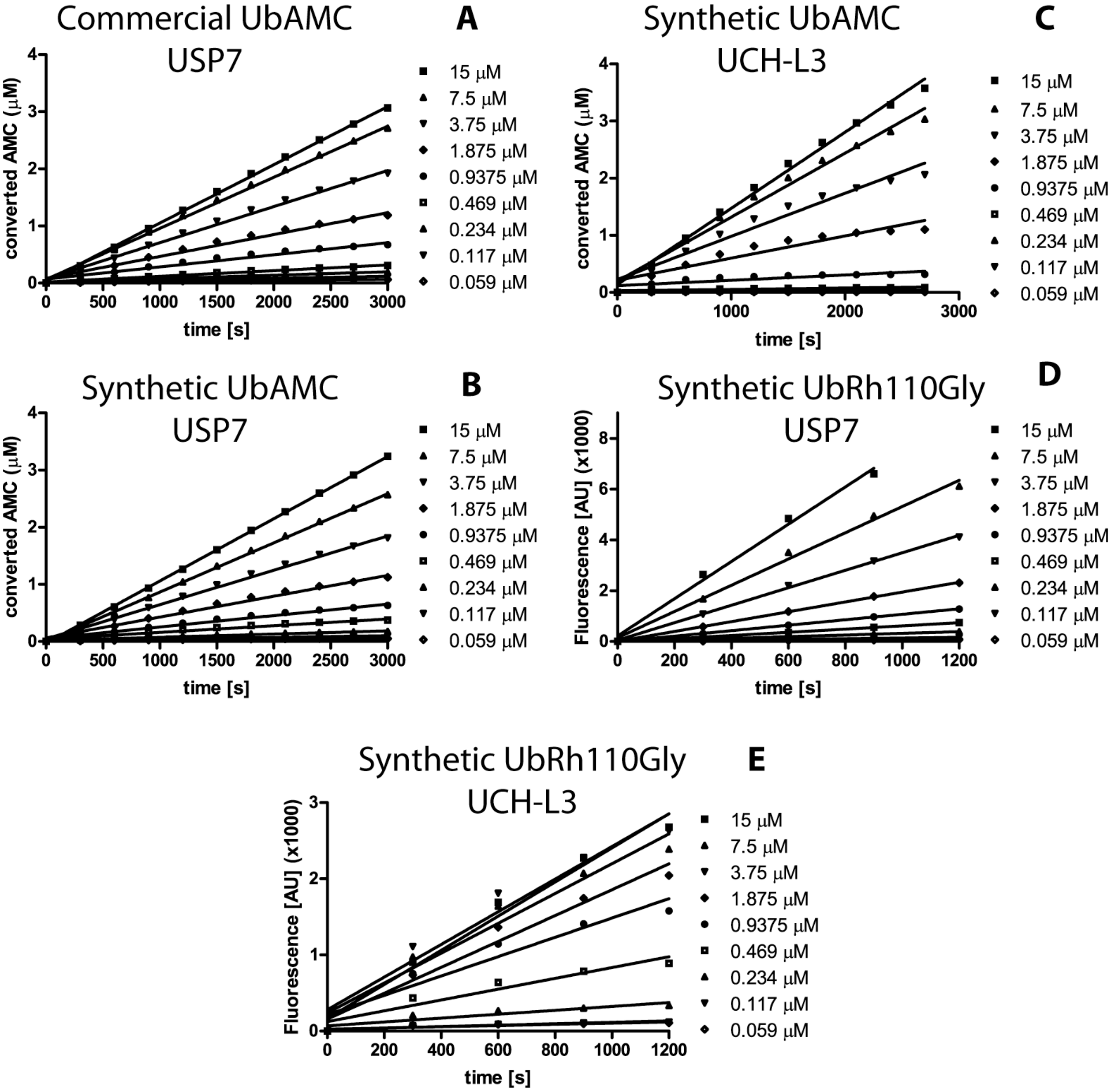

Protocol for Circular Dichroism experiment

CD spectra were measured in 5 mM NH4OAc (pH 6.5) at a concentration of 0.5 mg/ml. Spectra were measured using a custom build machine with 0.5 mm optical path length. Data was obtained by averaging 20 scans. Step size was 1 nm with 2 sec. acquisition time.

CD Spectrum

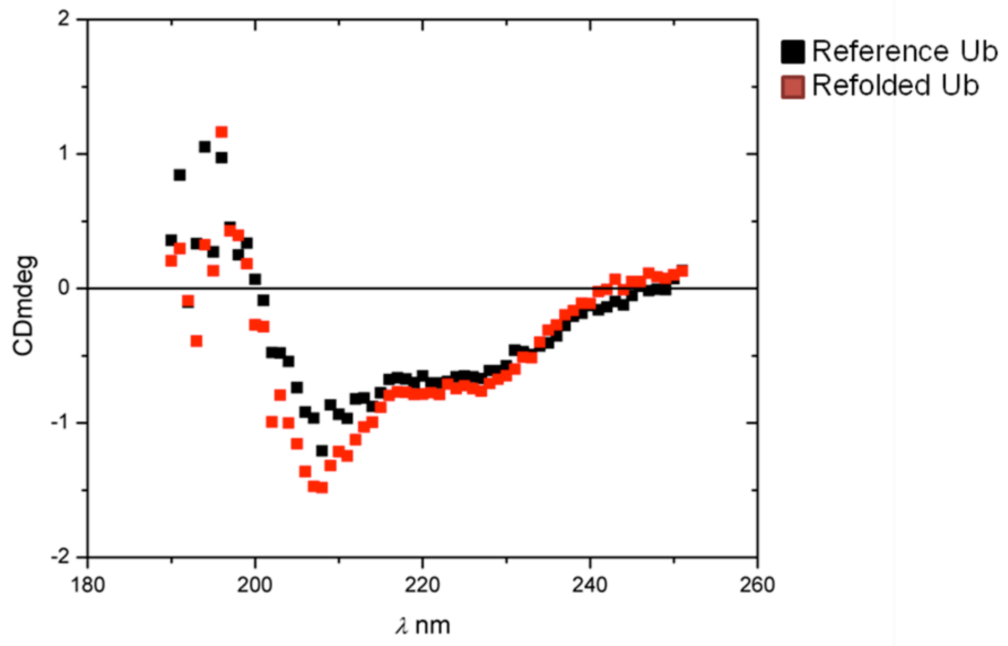

Figure S3B. Circular dichroism measurement of native ubiquitin (black) versus DMSO-folded ubiquitin (red).

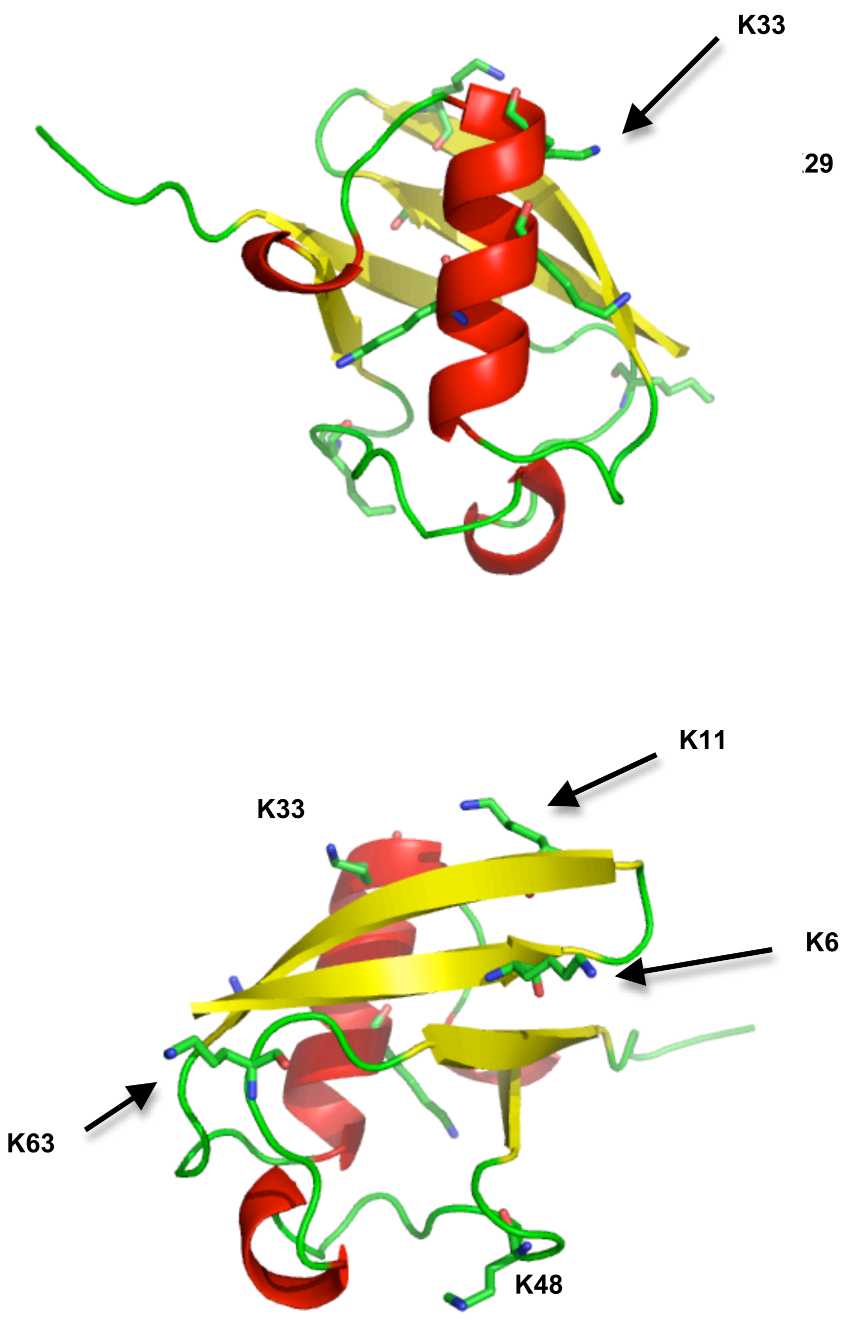

Figure S4. Positions of lysine residues in Ub (pdb code 1Ubi). DiUb ligation between Ub and δ-thiolysineG76V mutant under native conditions (under native condition using E1 mediated formation of Ub thioester) proved difficult in the case of the K27 and K29 δ-thiolysine mutant. This may be attributed to the more shielded environment of these 2 lysine residues (graphic generated with PyMOL Molecular Graphics System, Schrödinger, LLC).

LC-MS data

Ubiquitin K6δ-ThiolysineG76V

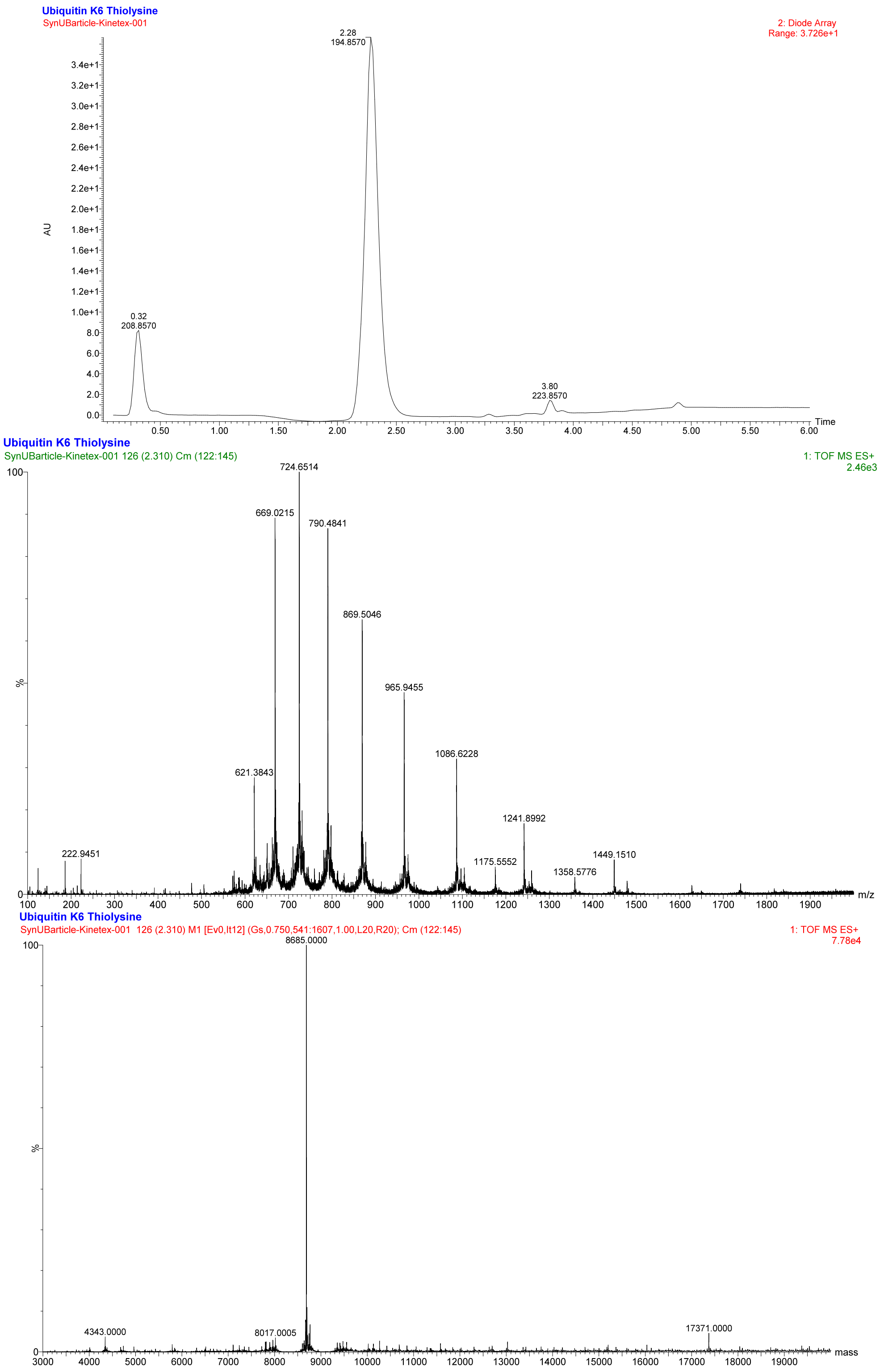

Figure S5. Ubiquitin with Lys6 mutated to δ-thiolysine and C-terminal Gly76 to Valine mutations. (top) Diode Array chromatogram using LC-MS method 3 (middle) Spectrum of peak at 2.28 min (bottom) Deconvoluted mass of peak found in middle *spectrum*. ESI-Mass [M+H] Expected: 8685.6/Found: 8685.

Ubiquitin K11δ-ThiolysineG76V

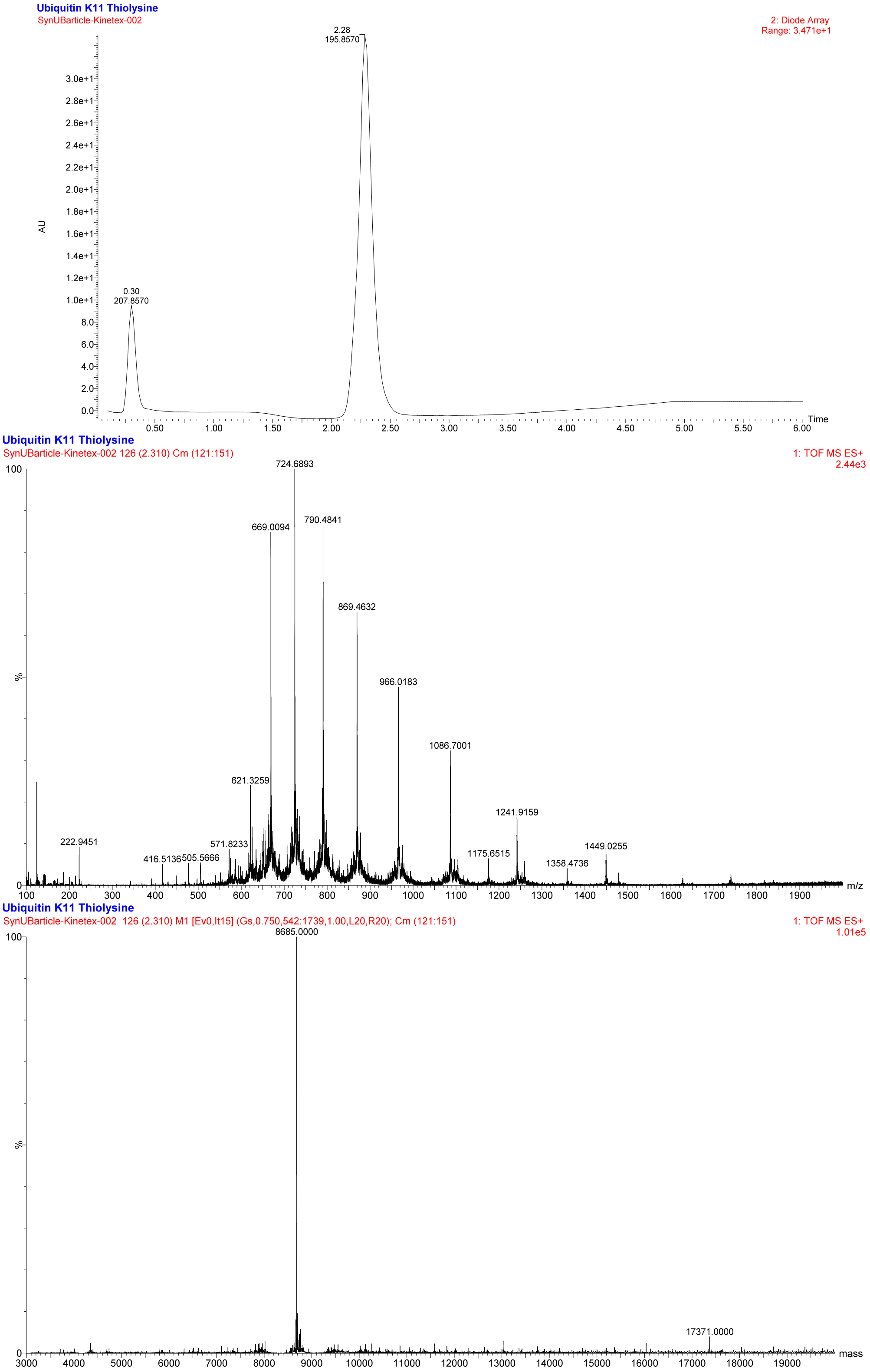

Figure S6. Ubiquitin with Lys11 mutated to δ-thiolysine and C-terminal Gly76 to Valine mutations. (top) Diode Array chromatogram using LC-MS method 3 (middle) Spectrum of peak at 2.28 min (bottom) Deconvoluted mass of peak found in middle *spectrum*. ESI-Mass [M+H] Expected: 8685.6/Found: 8685.

Ubiquitin K27δ-ThiolysineG76V

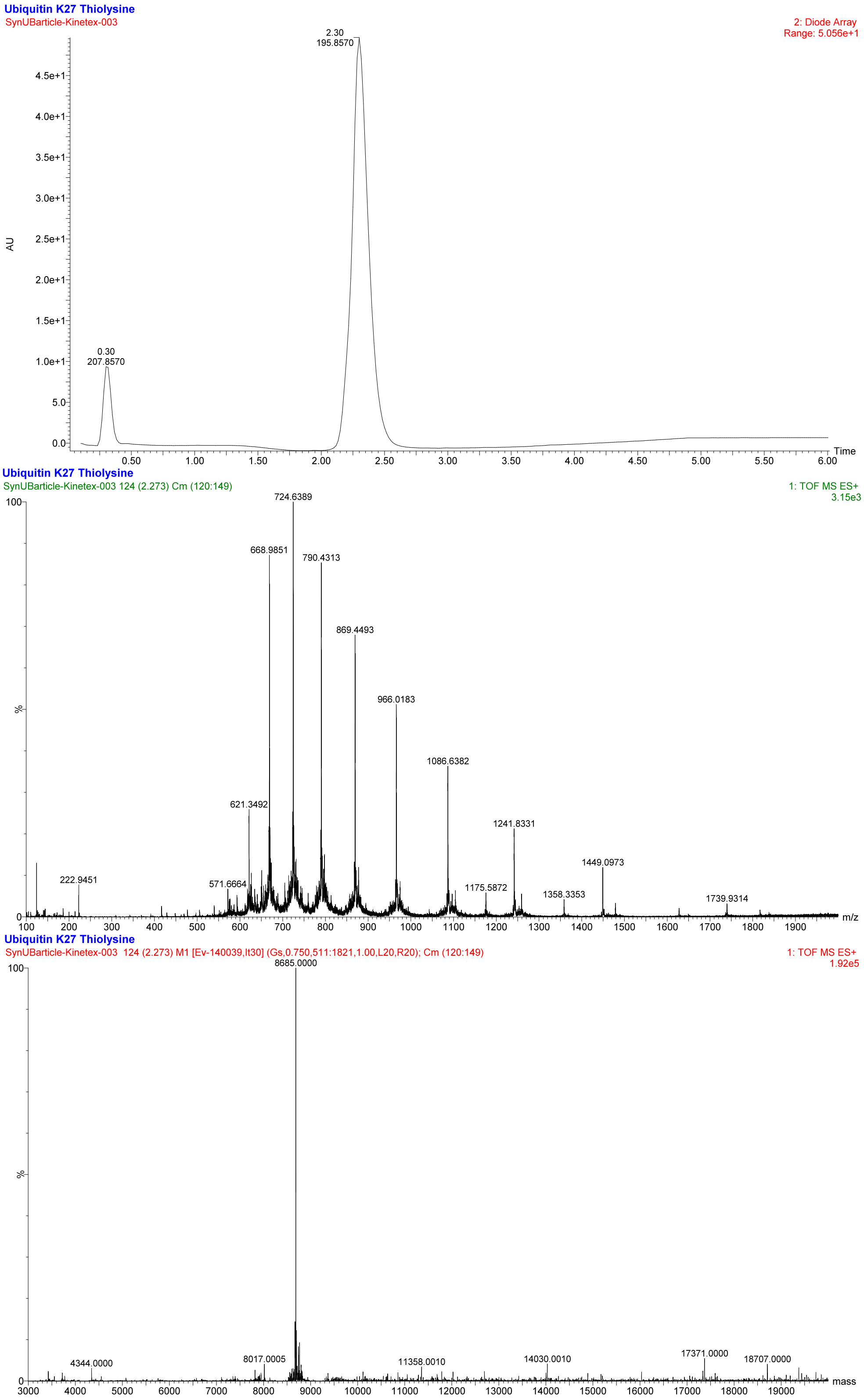

Figure S7. Ubiquitin with Lys27 mutated to δ-thiolysine and C-terminal Gly76 to Valine mutations. (top) Diode Array chromatogram using LC-MS method 3 (middle) Spectrum of peak at 2.30 min (bottom) Deconvoluted mass of peak found in middle *spectrum*. ESI-Mass [M+H] Expected: 8685.6/Found: 8685.

Ubiquitin K29δ-ThiolysineG76V

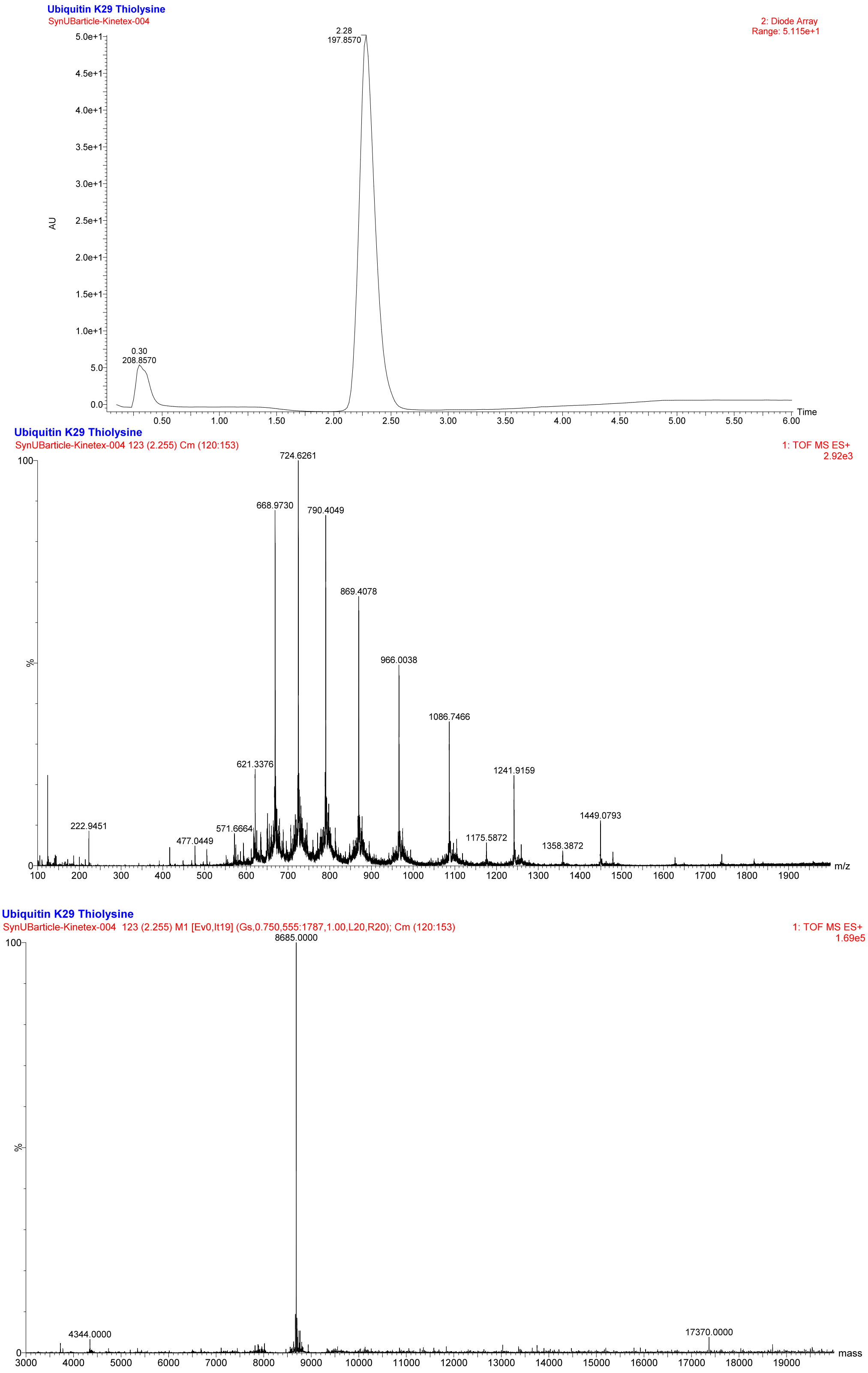

Figure S8. Ubiquitin with Lys29 mutated to δ-thiolysine and C-terminal Gly76 to Valine mutations. (top) Diode Array chromatogram using LC-MS method 3 (middle) Spectrum of peak at 2.28 min (bottom) Deconvoluted mass of peak found in middle *spectrum*. ESI-Mass [M+H] Expected: 8685.6/Found: 8685.

Ubiquitin K33δ-ThiolysineG76V

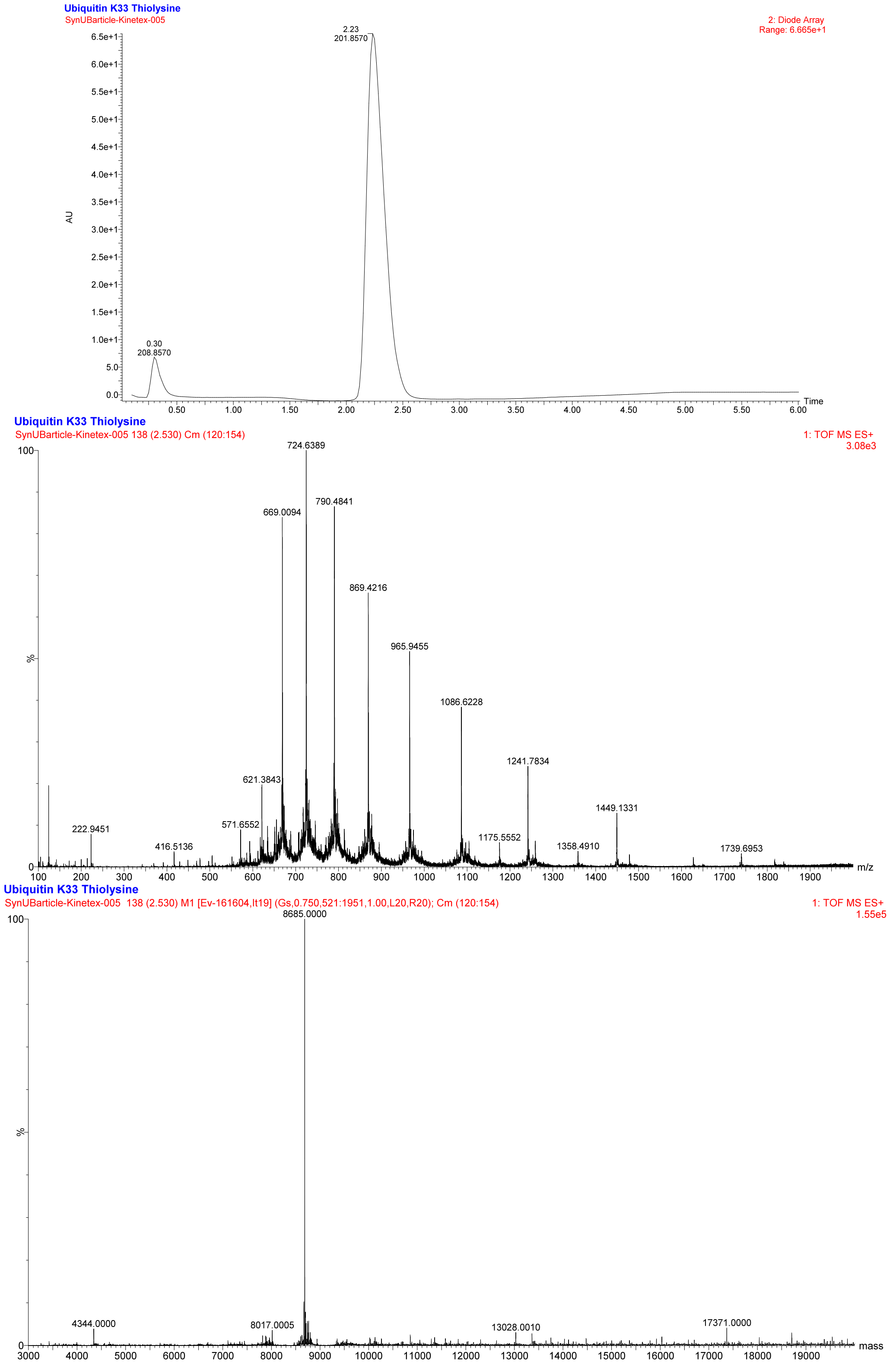

Figure S9. Ubiquitin with Lys33 mutated to δ-thiolysine and C-terminal Gly76 to Valine mutations. (top) Diode Array chromatogram using LC-MS method 3 (middle) Spectrum of peak at 2.23 min (bottom) Deconvoluted mass of peak found in middle *spectrum*. ESI-Mass [M+H] Expected: 8685.6/Found: 8685.

Ubiquitin K48δ-ThiolysineG76V

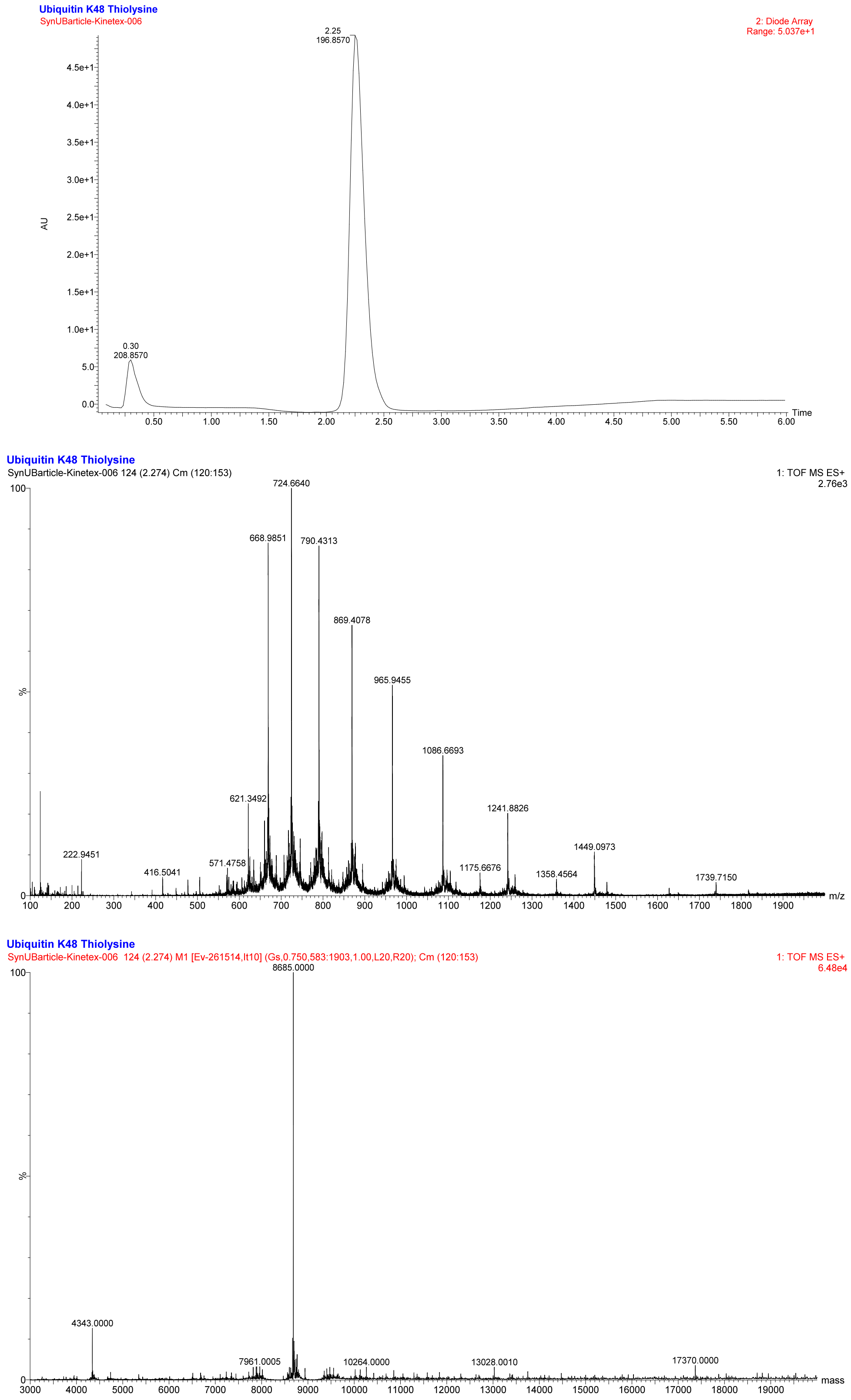

Figure S10. Ubiquitin with Lys48 mutated to δ-thiolysine and C-terminal Gly76 to Valine mutations. (top) Diode Array chromatogram using LC-MS method 3 (middle) Spectrum of peak at 2.25 min (bottom) Deconvoluted mass of peak found in middle *spectrum*. ESI-Mass [M+H] Expected: 8685.6/Found: 8685.

Ubiquitin K63δ-ThiolysineG76V

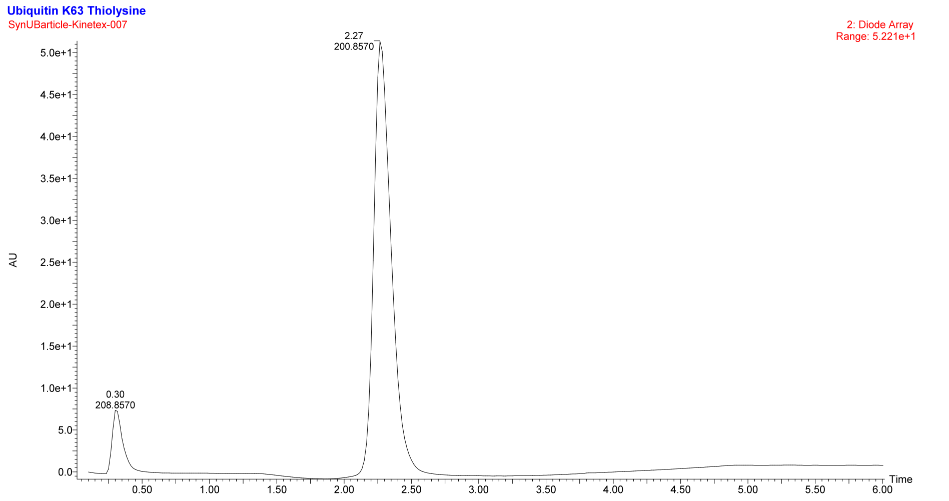

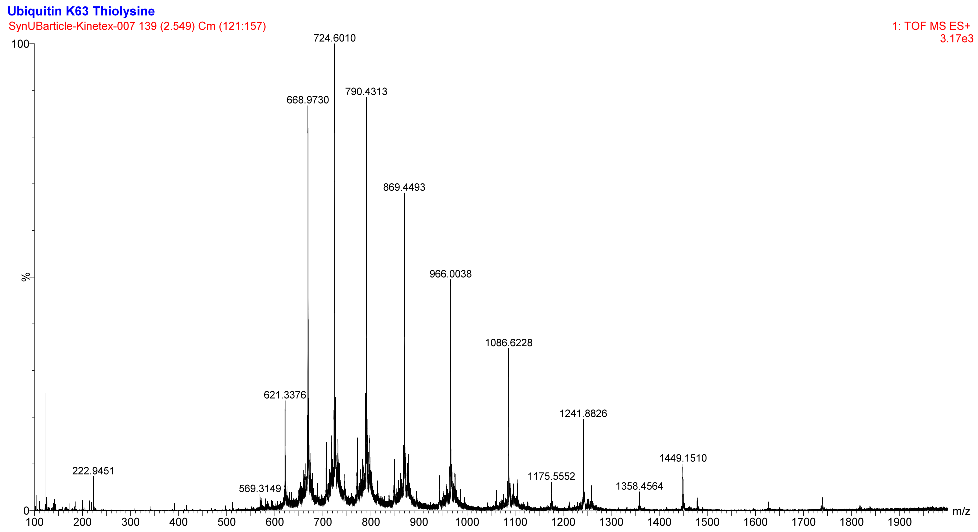

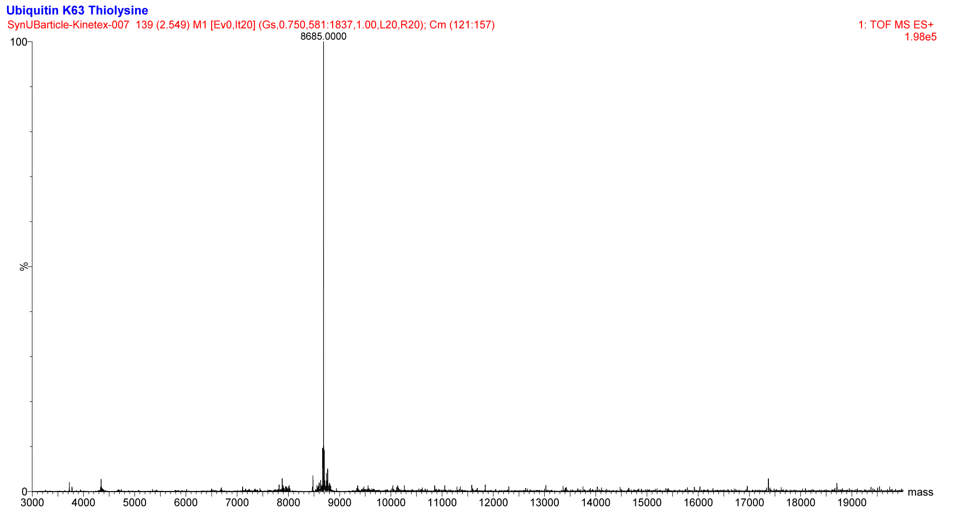

Figure S11. Ubiquitin with Lys63 mutated to 5-thiolysine and C-terminal Gly76 to Valine mutations. (top) Diode Array chromatogram using LC-MS method 3 (middle) Spectrum of peak at 2.27 min (bottom) Deconvoluted mass of peak found in middle *spectrum*. ESI-Mass [M+H] Expected: 8685.6/Found: 8685.

Ubiquitin Rhodamine110

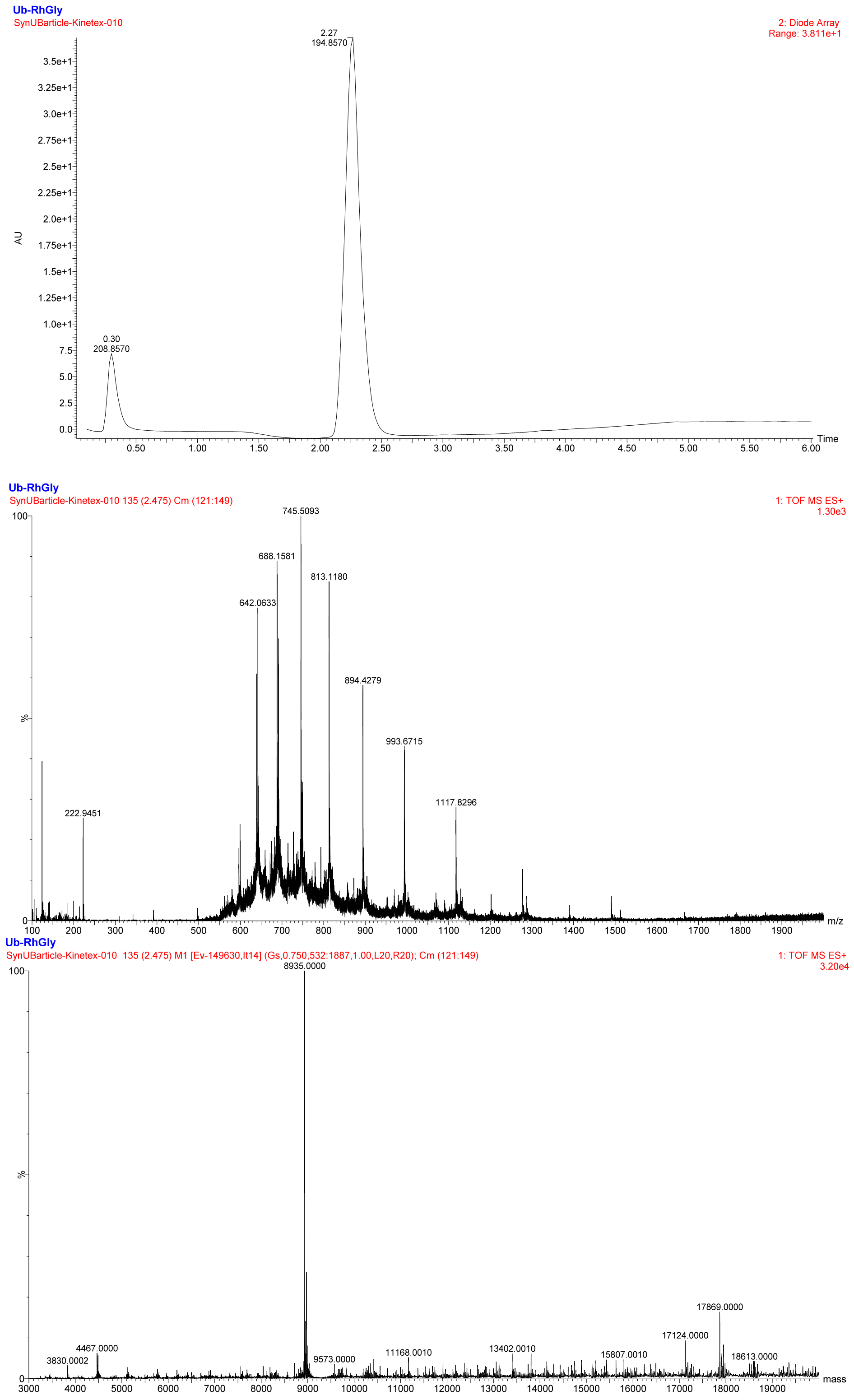

Figure S13. Ub with C-terminal Gly75 coupled to GlyRh110Gly. (top) Diode Array chromatogram using LC-MS method 3 (middle) Spectrum of peak at 2.27 min (bottom) Deconvoluted mass of peak found in middle *spectrum*. ESI-Mass [M+H] Expected: 8934.8/Found: 8935.

Ubiquitin-AMC

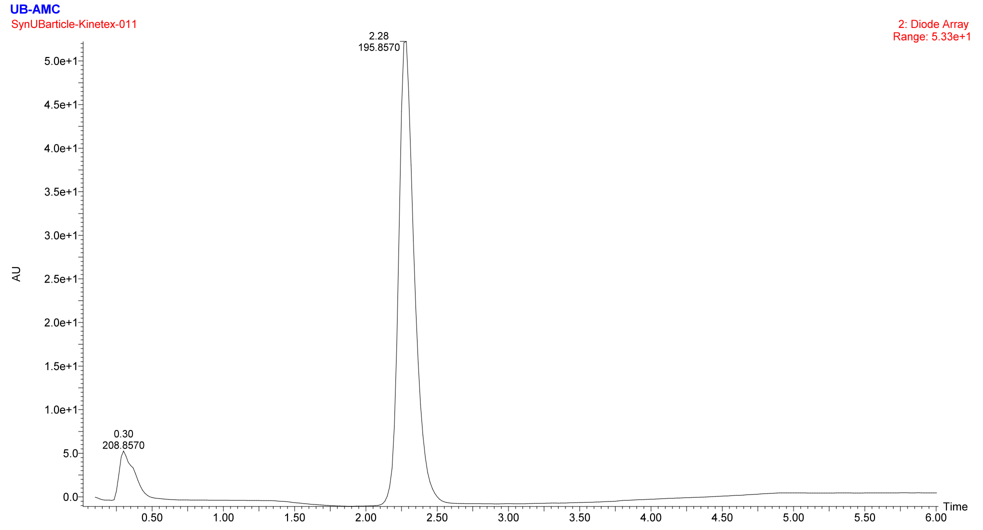

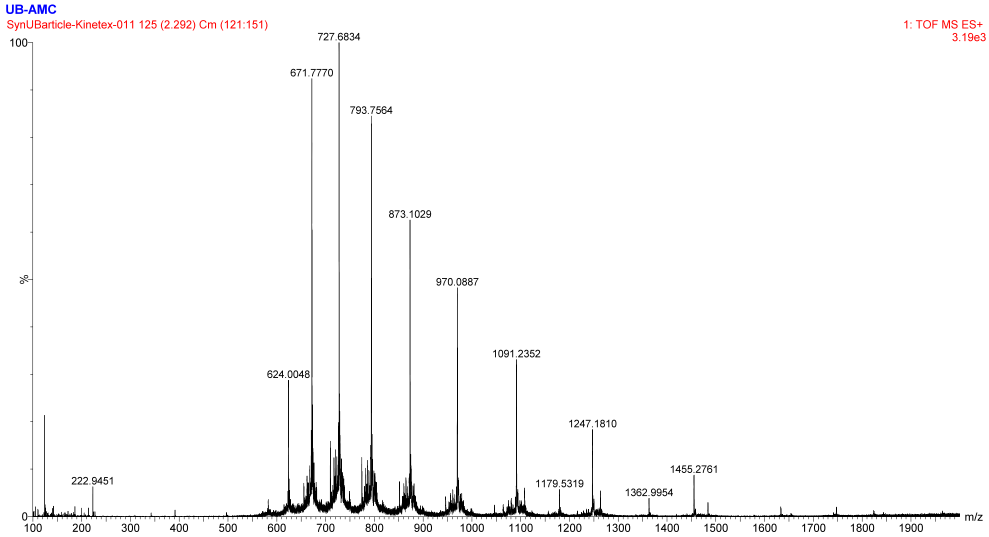

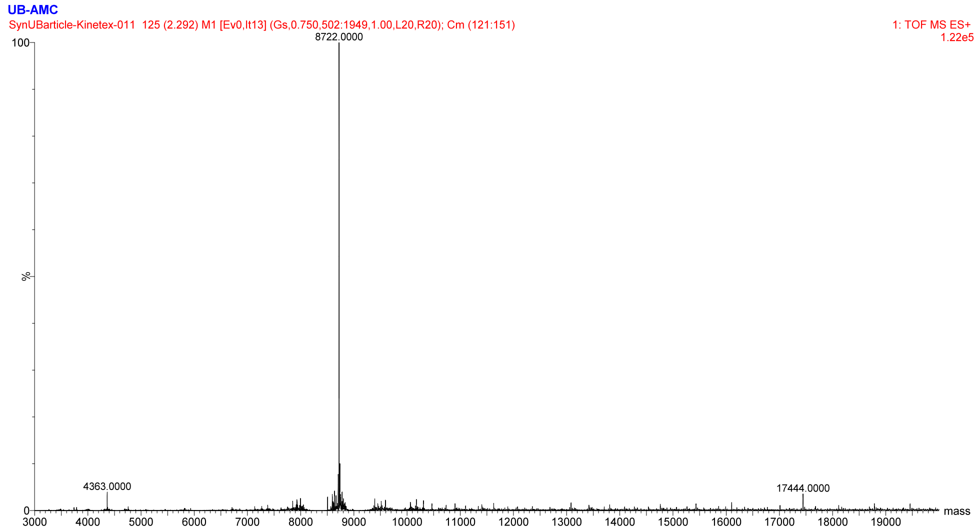

Figure S14. Ub with 7-amido-4-methyl coumarin coupled to its Gly76 *C*-terminus. (top) Diode Array chromatogram using LC-MS method 3 (middle) Spectrum of peak at 2.28 min (bottom) Deconvoluted mass of peak found in middle *spectrum*. ESI-Mass [M+H] Expected: 8722.6/Found: 8722.

His$_6$-Ubiquitin

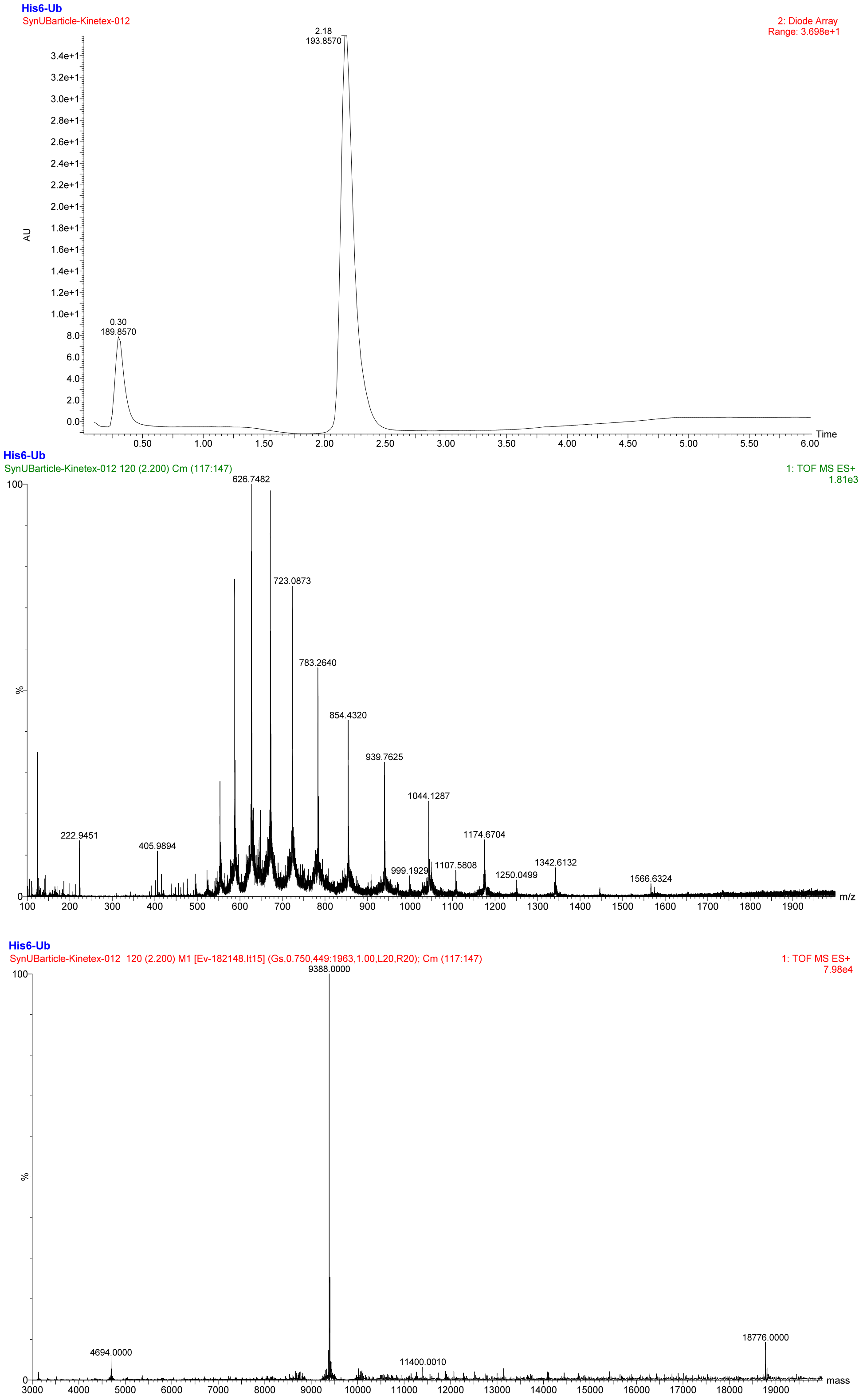

Figure S15. Ubiquitin with HHHHHH motif on *N*-terminus. (top) Diode Array chromatogram using LC-MS method 3 (middle) Spectrum of peak at 2.18 min (bottom) Deconvoluted mass of peak found in middle *spectrum*. ESI-Mass [M+H] Expected: 9388.19/Found: 9388.

HA-Ubiquitin

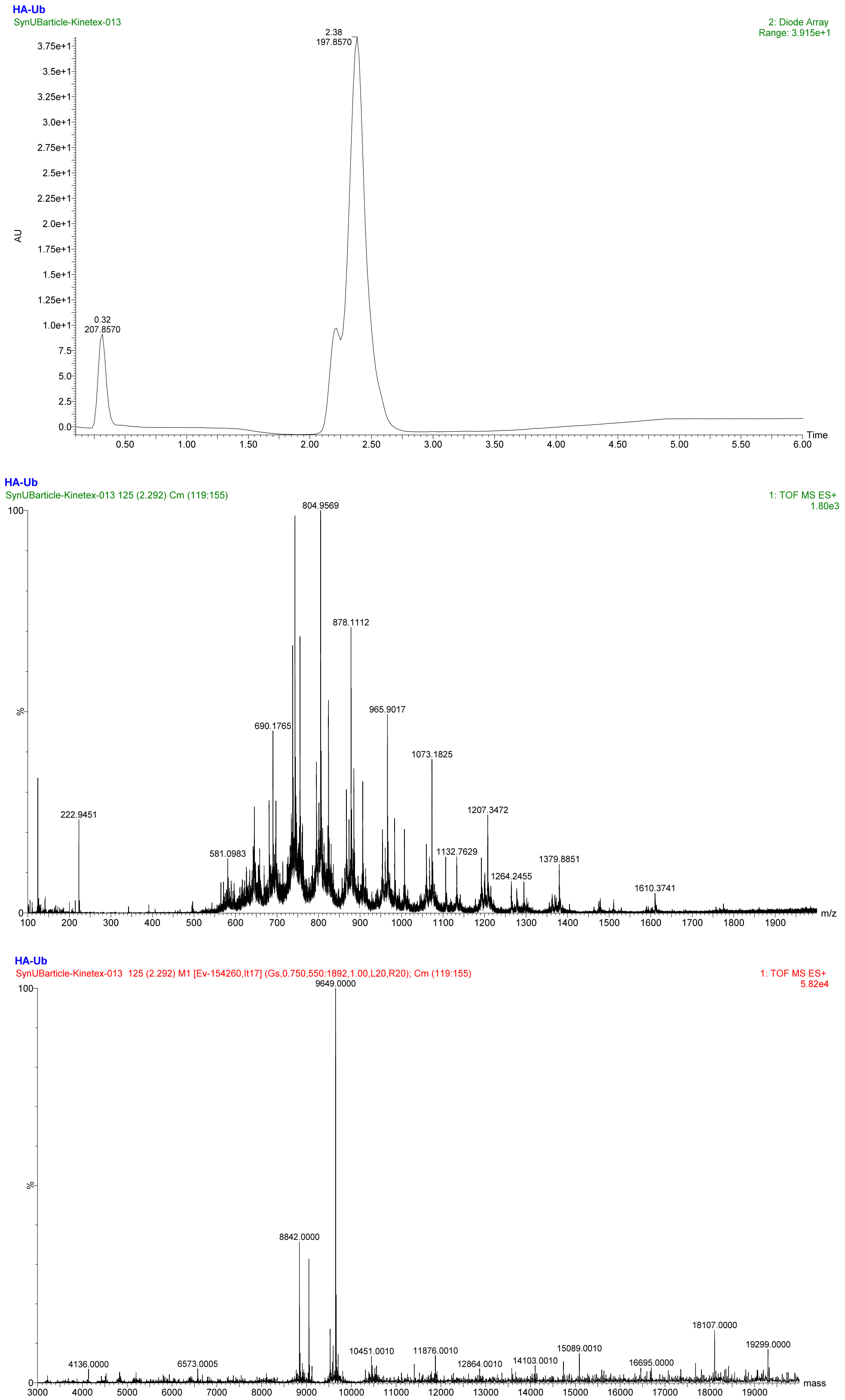

Figure S16. Ubiquitin with YPYDVPDYA motif (HA-tag) on *N*-terminus. (top) Diode Array chromatogram using LC-MS method 3 (middle) Spectrum of peak at 2.28 min (bottom) Deconvoluted mass of peak found in middle *spectrum*. ESI-Mass [M+H] Expected: 9649.5/Found: 9649.

DOTA-Ubiquitin

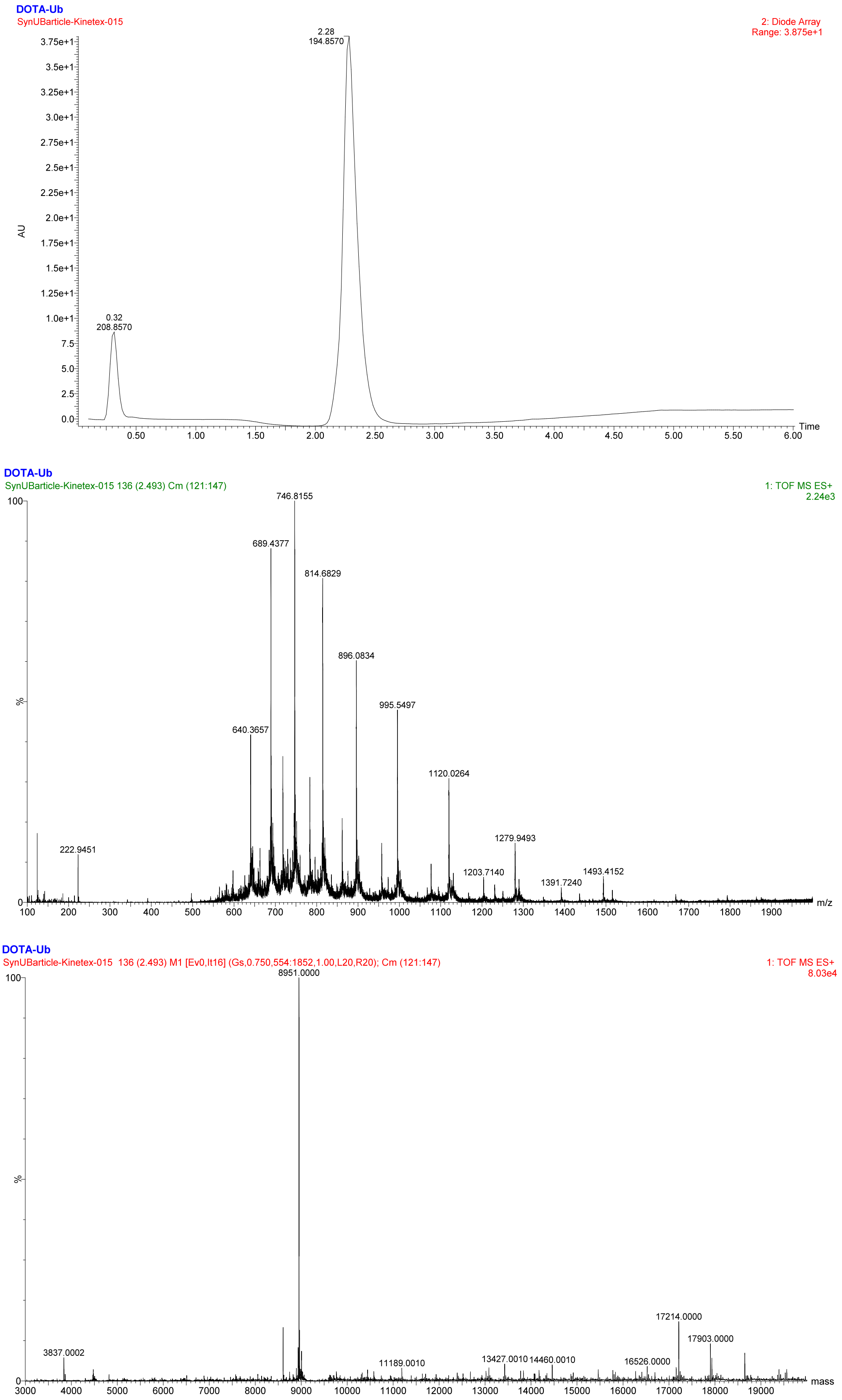

Figure S18. Ubiquitin with DOTA-tag on *N*-terminus. (top) Diode Array chromatogram using LC-MS method 3 (middle) Spectrum of peak at 2.28 min (bottom) Deconvoluted mass of peak found in middle *spectrum*. ESI-Mass [M+H] Expected: 8951.8 /Found: 8951.

Tetramethylrhodamine Ubiquitin

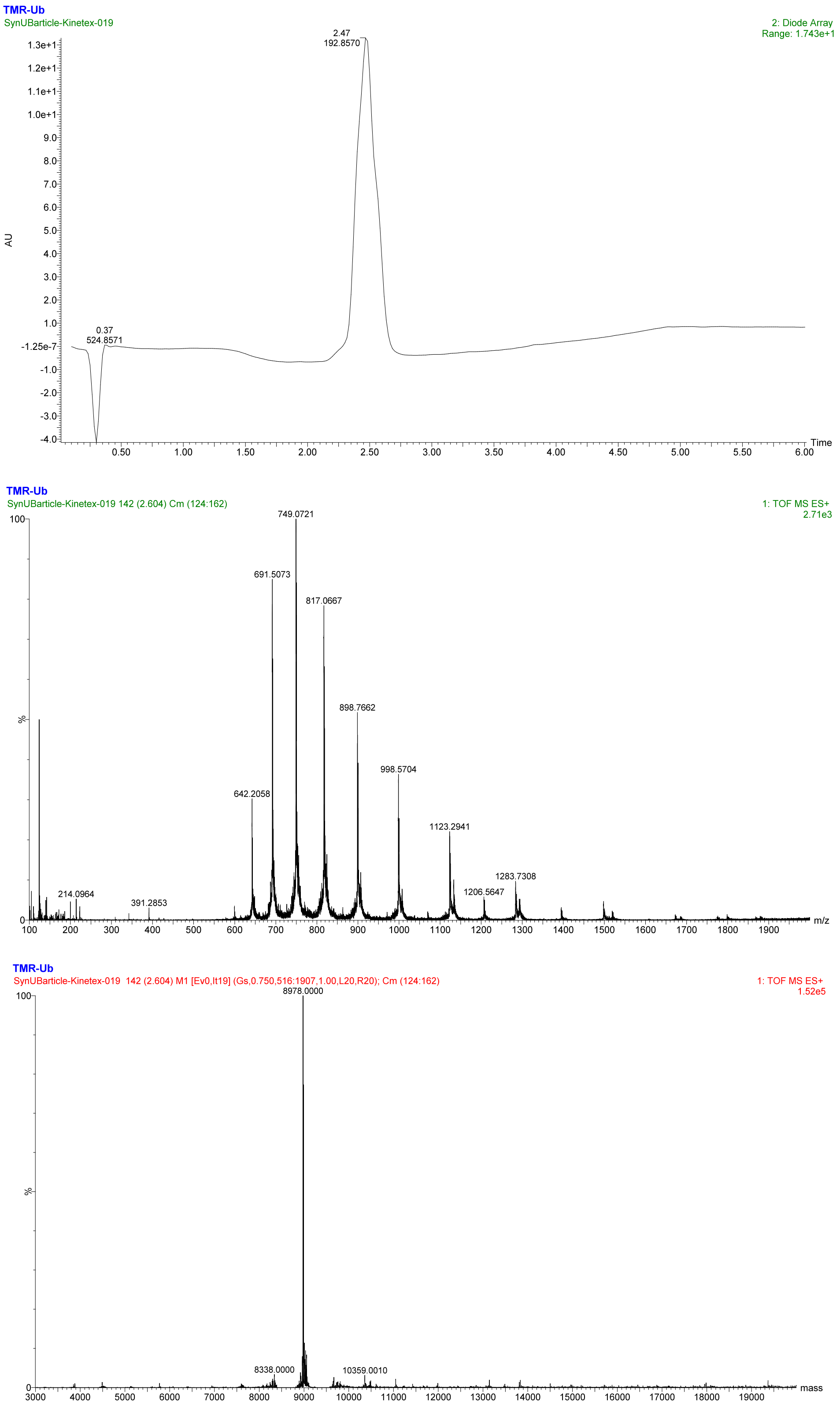

Figure S19. Ubiquitin with Tetramethylrhodamine on *N*-terminus. (top) Diode Array chromatogram using LC-MS method 3 (middle) Spectrum of peak at 2.38 min (bottom) Deconvoluted mass of peak found in middle *spectrum*. ESI-Mass [M+H] Expected: 8977.8/Found: 8978.

Ubiquitin K to δ-Thiolysine Mutants

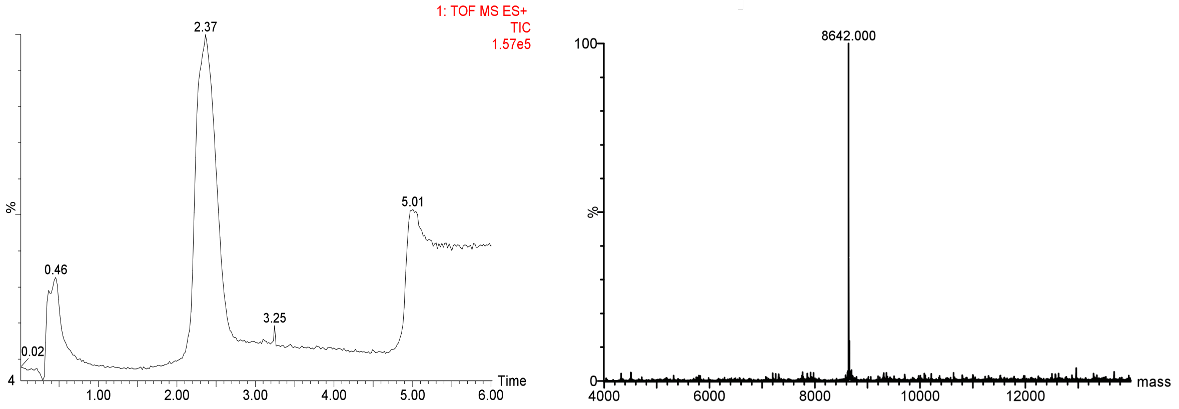

Figure S20. (A) LC-MS analysis Ub with Lys6 mutated to δ-thiolysine. (left) MS chromatogram using LC-MS method 3. (right) Deconvoluted mass of product peak at 2.4 min. ESI-Mass [M+H] Expected: 8643 Da; found: 8642 Da.

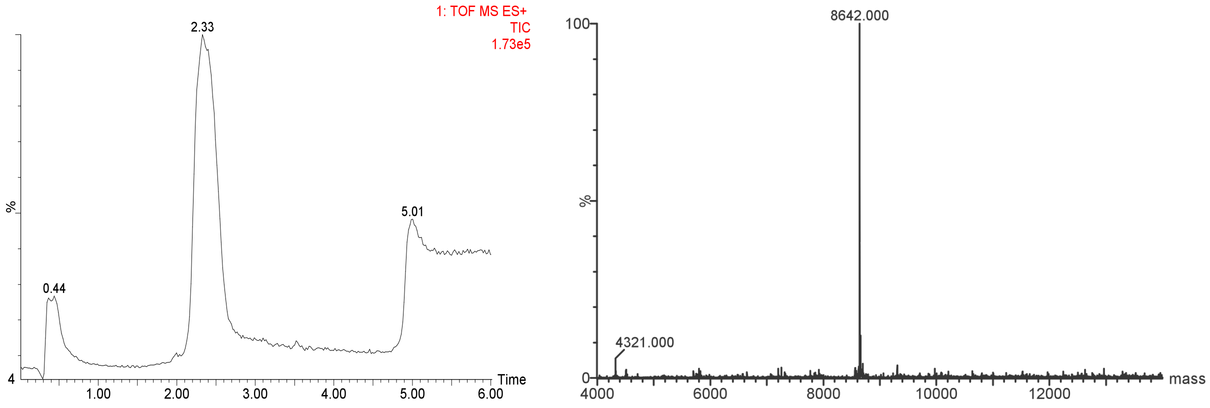

Figure S21. (A) LC-MS analysis Ub with Lys11 mutated to δ-thiolysine. (left) MS chromatogram using LC-MS method 3. (right) Deconvoluted mass of product peak at 2.3 min. ESI-Mass [M+H] Expected: 8643 Da; found: 8642 Da.

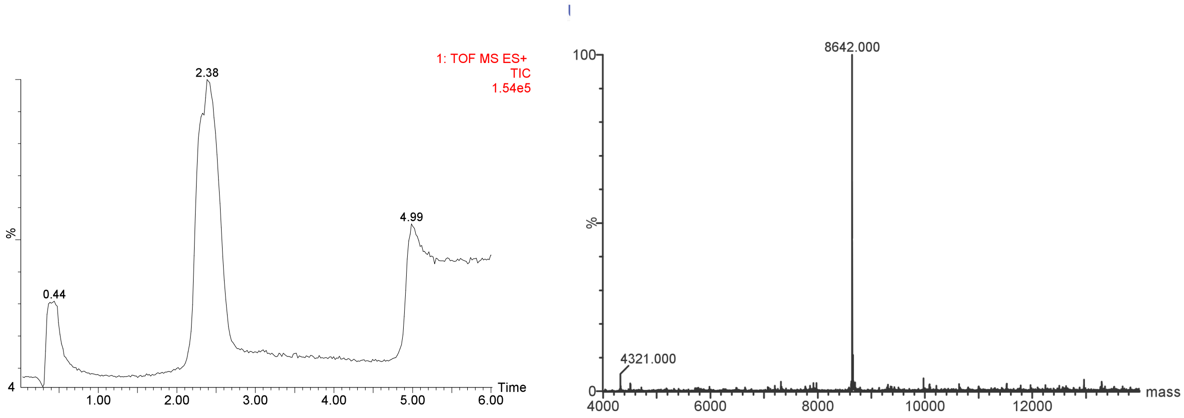

Figure S22. (A) LC-MS analysis Ub with Lys27 mutated to δ-thiolysine. (left) MS chromatogram using LC-MS method 3. (right) Deconvoluted mass of product peak at 2.4 min. ESI-Mass [M+H] Expected: 8643 Da; found: 8642 Da.

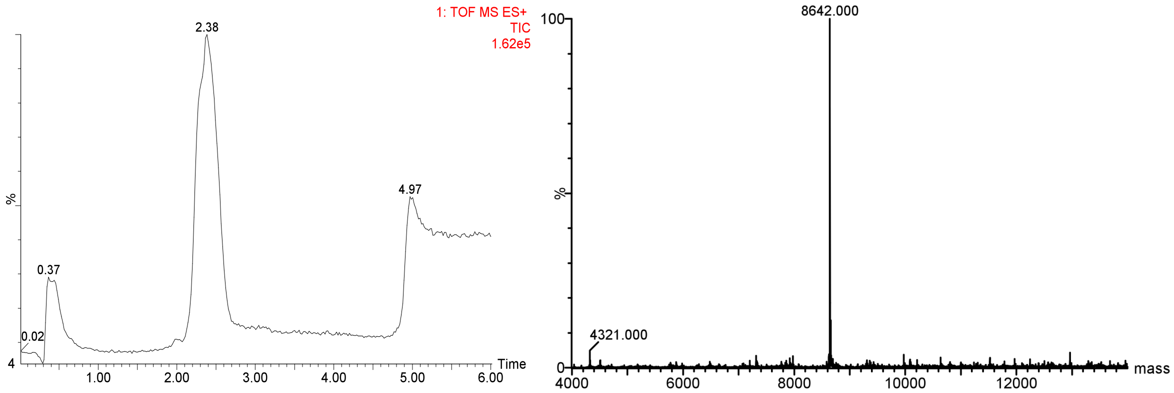

Figure S23. (A) LC-MS analysis Ub with Lys29 mutated to δ-thiolysine. (left) MS chromatogram using LC-MS method 3. (right) Deconvoluted mass of product peak at 2.4 min. ESI-Mass [M+H] Expected: 8643 Da; found: 8642 Da.

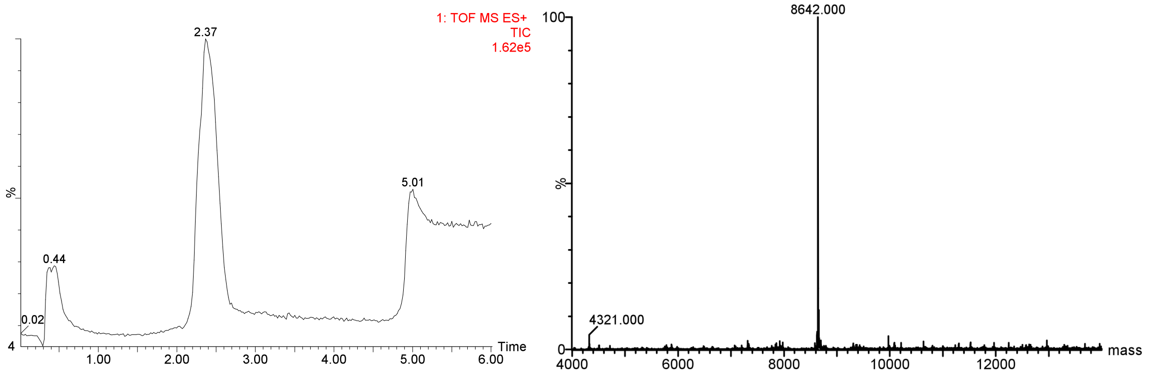

Figure S24. (A) LC-MS analysis Ub with Lys33 mutated to δ-thiolysine. (left) MS chromatogram using LC-MS method 3. (right) Deconvoluted mass of product peak at 2.4 min. ESI-Mass [M+H] Expected: 8643 Da; found: 8642 Da.

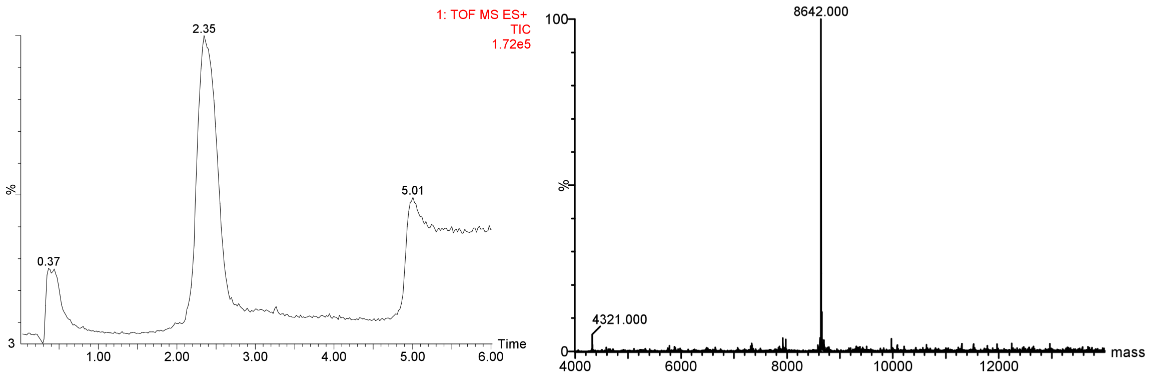

Figure S25. (A) LC-MS analysis Ub with Lys48 mutated to δ-thiolysine. (left) MS chromatogram using LC-MS method 3. (right) Deconvoluted mass of product peak at 2.4 min. ESI-Mass [M+H] Expected: 8643 Da; found: 8642 Da.

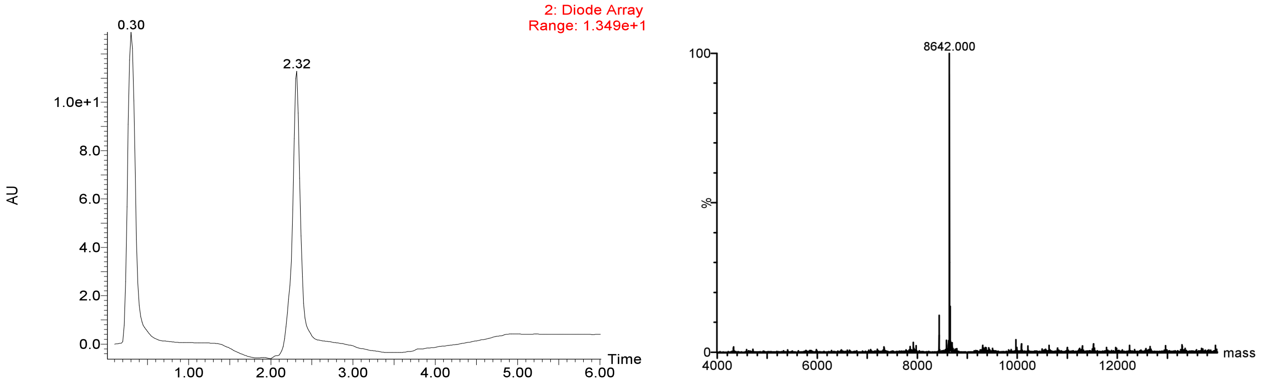

Figure S26. (A) LC-MS analysis Ub with Lys63 mutated to δ-thiolysine. MS chromatogram (top left) and UV-trace (top right) using LC-MS method 3. (right) Deconvoluted mass of product peak at 2.4 min. ESI-Mass [M+H] Expected: 8643 Da; found: 8642 Da.

Chemoenzymatic UbMESNa synthesis

Full-length UbMESNa was formed by incubating Ub (100 μM) for 5−6 h at 37°C with 250 nM E1, 10 mM $MgCl_2$, 10 mM ATP and 100 mM MESNa in 50 mM sodium phosphate buffer pH 8.0. The product was purified by RP-HPLC, lyophilized and stored at room temperature. We have performed this reaction on a 10 mg and 40 mg scale, isolating in both cases UbMESNa in ±80% yield after RP-HPLC.

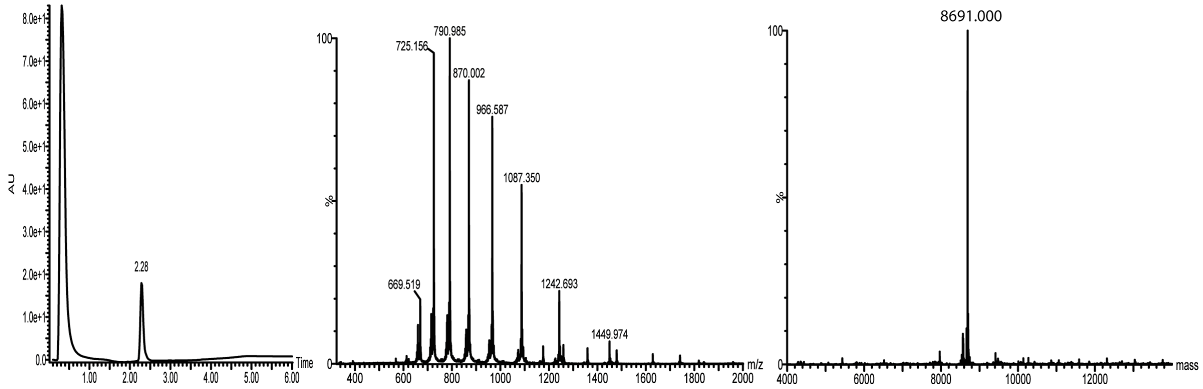

Figure S27. Analysis of purified UbMESNa.

Protocol for ligations between Ub and UbK to δ-thiolysine/G76V mutants under native conditions. A solution of Ub (100 µM) and Ub thiolysine mutant (100 µM) in 50 mM sodium phosphate buffer pH 8 was incubated at 37°C with 500 nM E1, 10 mM $MgCl_2$, 10 mM ATP, 50 mM 4-MPAA and 50 mM TCEP. Besides 50 mM MPAA, a mixture of MPAA (25 mM) and MESNa (25 mM) also gave satisfactory results.

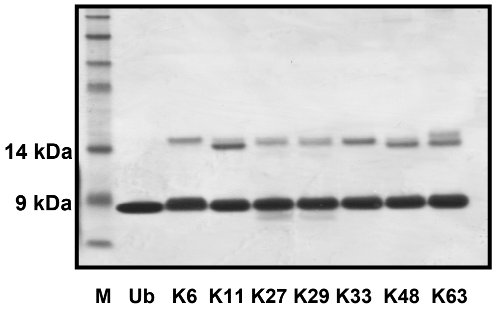

Figure S28. Initial set of DiUb ligations under denaturing conditions using Ub concentrations of 100 µM.

DiUbiquitin ligation under denaturing conditions. Initial small scale reactions under denaturing reaction conditions (6M Gdn·HCl pH 7.5) uses 100 mM MPAA, 50 mM TCEP with input ubiquitin thioester and mutant at a concentration of 100 µM in a 1:1 ratio, resulted in the formation of all desired topoisomers, as judged by SDS-PAGE (Figure S28) and LC-MS analysis (ligation products were observed in all cases as diUb MPAA disulfides). Based on these results we devised the following preparative scale diUb ligation protocol:
A solution of Ub (1.15 mM) and Ub δ-thiolysine mutant (1.15 mM) in 6M Gn•HCl (pH 8) was incubated at 37°C with 100 mM 4-MPAA and 50 mM TCEP. After overnight incubation, a fresh portion of UbMESNa (0.5 eq) was added and the reaction incubated overnight at 37°C. Ligations were analysed by LC-MS (program 3) and SDS-PAGE (12% bis-tris precast gel, MES buffer). The reaction mixture was purified by RP-HPLC as follows: mobile phases: A = 0.05% TFA in MQ water and B = 0.05% TFA in CH_3CN. Waters Atlantis® dC_{18} OBD™ (19×250 mm, 10 µM); flow rate = 18 mL/min. Gradient: 0−1 min: 20% B; 1−3 min: 20% ⇨ 32% B; 3−23 min: 32% ⇨ 42% B; 23−24 min: 42% ⇨ 95% B. Yield of DiUb conjugates of UbLys to δ-thiolysine/G76V double mutant:

**K6 linked**	:	white powder, 8.5 mg, 59%	−	R_t 14.4 min
**K11 linked**	:	white powder, 10.8 mg, 72%	−	R_t 15.6 min
**K27 linked**	:	white powder, 9.6 mg, 64%	−	R_t 13.8 min
**K29 linked**	:	white powder, 8.0 mg, 35%	−	R_t 11.1 min
**K33 linked**	:	white powder, 9.4 mg, 62%	−	R_t 8.9 min
**K48 linked**	:	white powder, 9.4 mg, 64%	−	R_t 13.1 min
**K63 linked**	:	white powder, 8.0 mg, 53%	−	R_t 14.8 min

General protocol for desulfurization of diUb conjugates. To a solution of diUb conjugate in 6M Gdn•HCl, 0.1 M sodium phosphate pH 6.5 (0.2 mg/mL – 1 mg/mL) was added V-50 (final conc 200 mM), TCEP (final conc 0.25 M) and glutathione (final conc 40 mM). After adjusting the pH to 6.5 with 1N NaOH, the reaction was incubated overnight at 60°C (water bath) and analysed by LC-MS. HPLC purification was performed as described above.

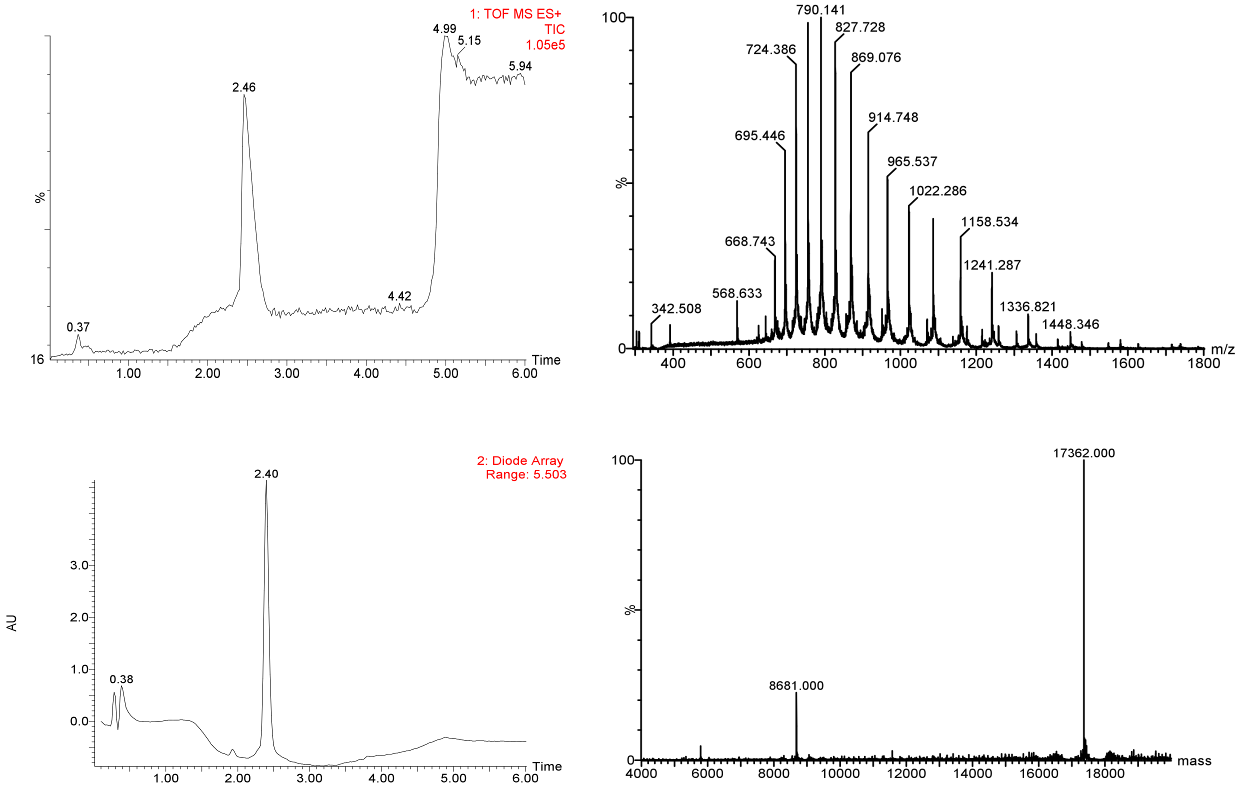

Figure S29. LC (top left) and MS analysis (top right) of K6-linked diUb conjugate (δ-thiolysine/G76V double mutant), LC-MS program 3. (bottom left): UV-trace. (bottom right): deconvoluted mass of product peak at 2.5 min.

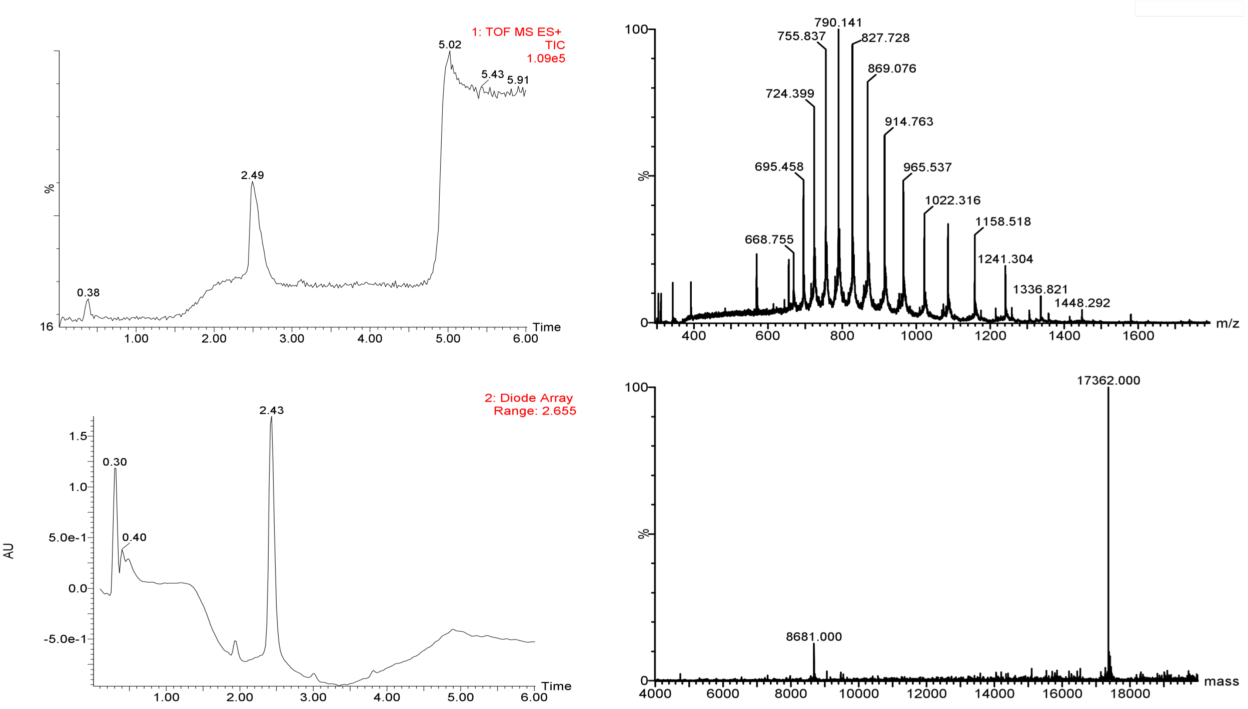

Figure S30. LC (top left) and MS analysis (top right) of K11-linked diUb conjugate (δ-thiolysine/G76V double mutant), LC-MS program 3. (bottom left): UV-trace. (bottom right): deconvoluted mass of product peak at 2.5 min.

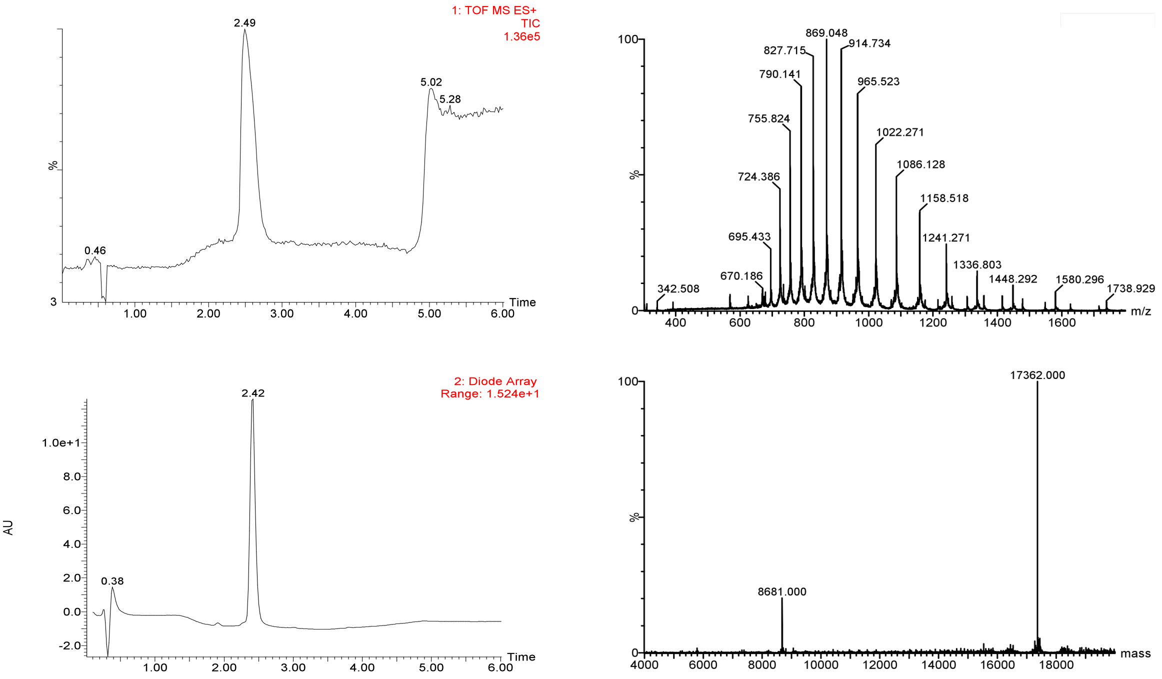

Figure S31. LC (top left) and MS analysis (top right) of K27-linked diUb conjugate (δ-thiolysine/G76V double mutant), LC-MS program 3. (bottom left): UV-trace. (bottom right): deconvoluted mass of product peak at 2.5 min.

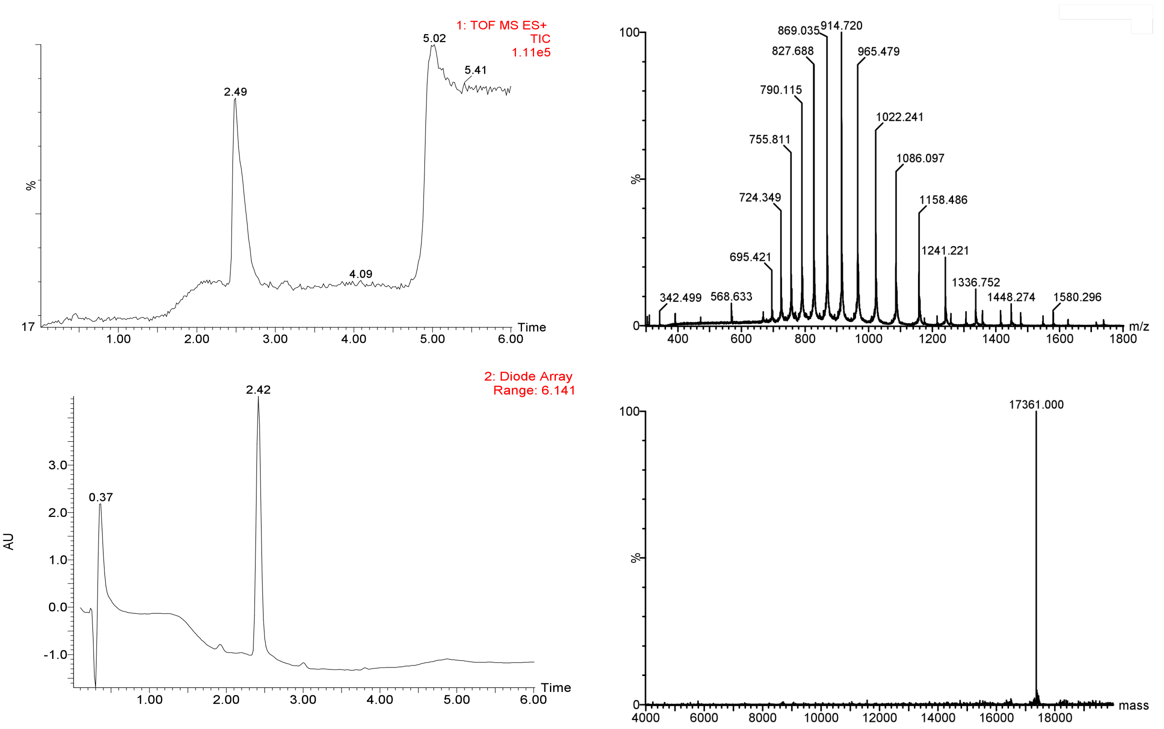

Figure S32. LC (top left) and MS analysis (top right) of K29-linked diUb conjugate (δ-thiolysine/G76V double mutant), LC-MS program 3. (bottom left): UV-trace. (bottom right): deconvoluted mass of product peak at 2.5 min.

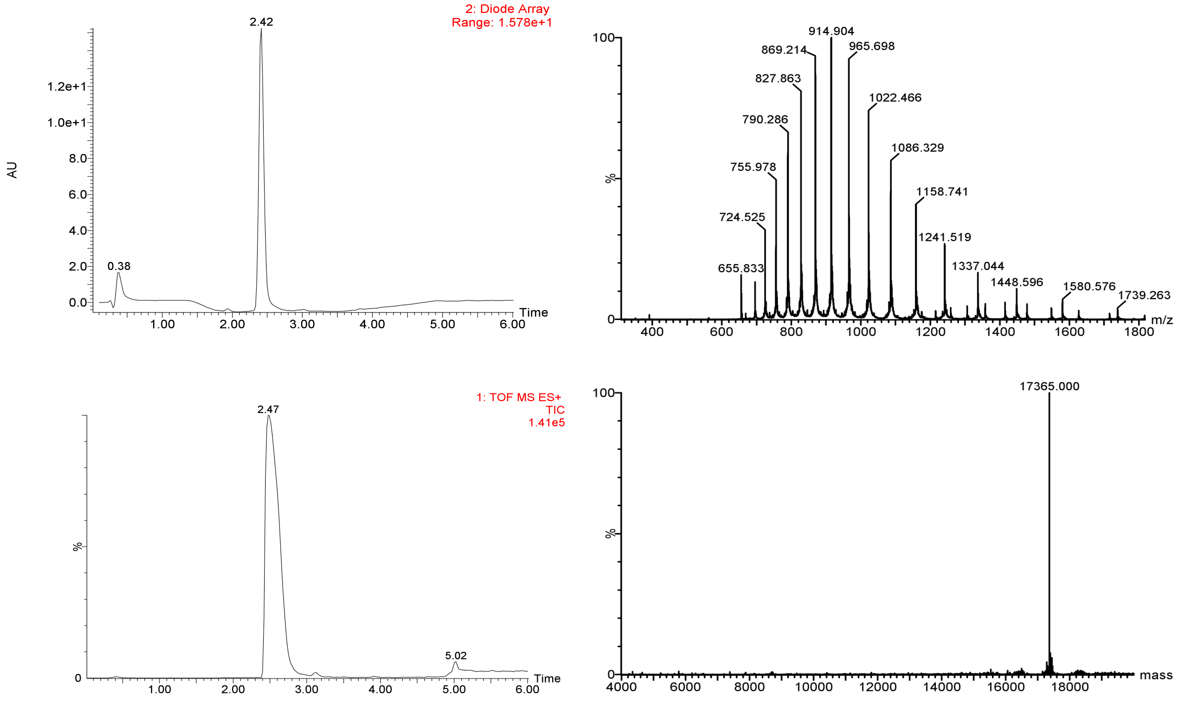

Figure S33. LC (top left) and MS analysis (top right) of K33-linked diUb conjugate (δ-thiolysine/G76V double mutant), LC-MS program 3. (bottom left): UV-trace. (bottom right): deconvoluted mass of product peak at 2.5 min.

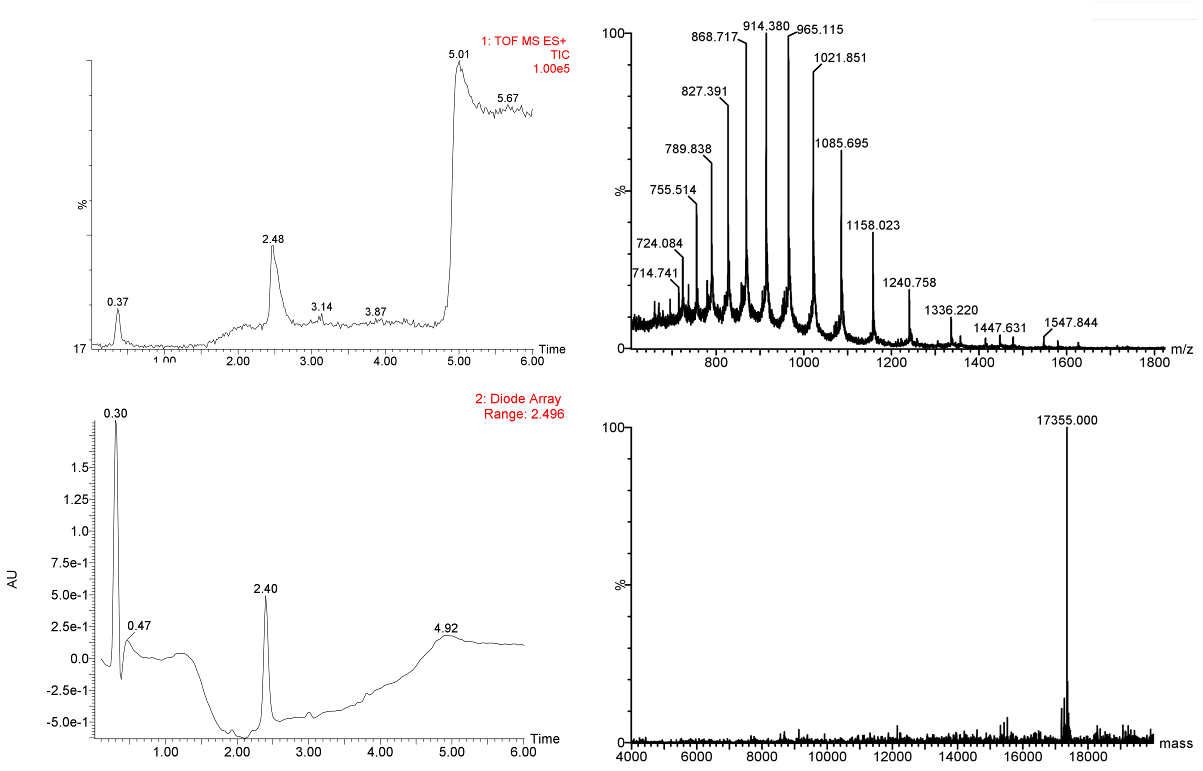

Figure S34. LC (top left) and MS analysis (top right) of K48-linked diUb conjugate (δ-thiolysine/G76V double mutant), LC-MS program 3. (bottom left): UV-trace. (bottom right): deconvoluted mass of product peak at 2.5 min.

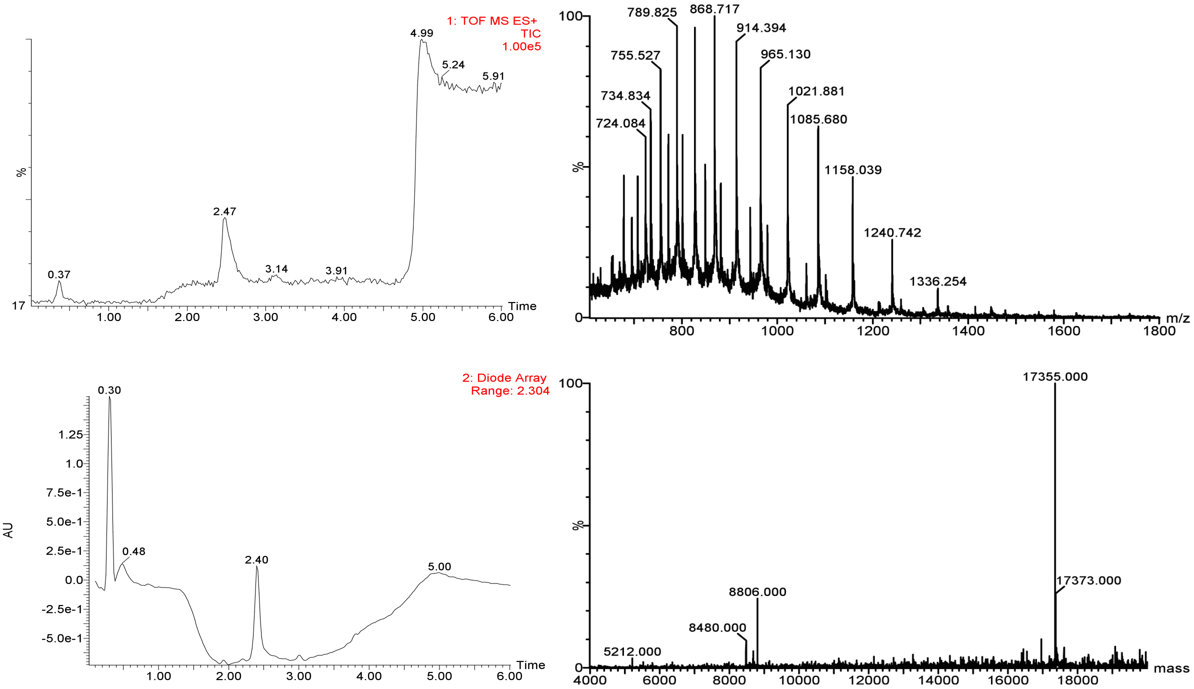

Figure S34. LC (top left) and MS analysis (top right) of K63-linked diUb conjugate (δ-thiolysine/G76V double mutant), LC-MS program 3. (bottom left): UV-trace. (bottom right): deconvoluted mass of product peak at 2.5 min.

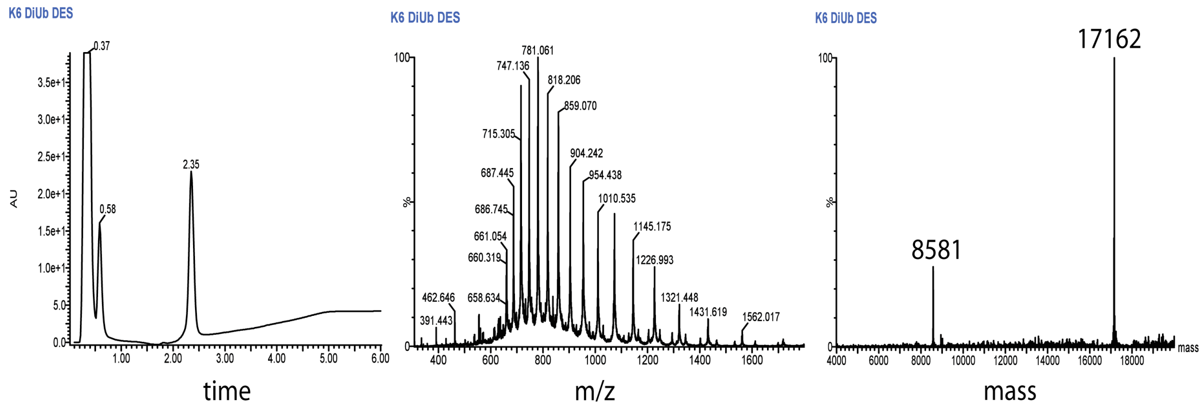

Figure S35. LC-MS analysis (program 3) of V-50 desulfurized K6-linked diUb conjugate (note that modified Ub has a G76V mutation).

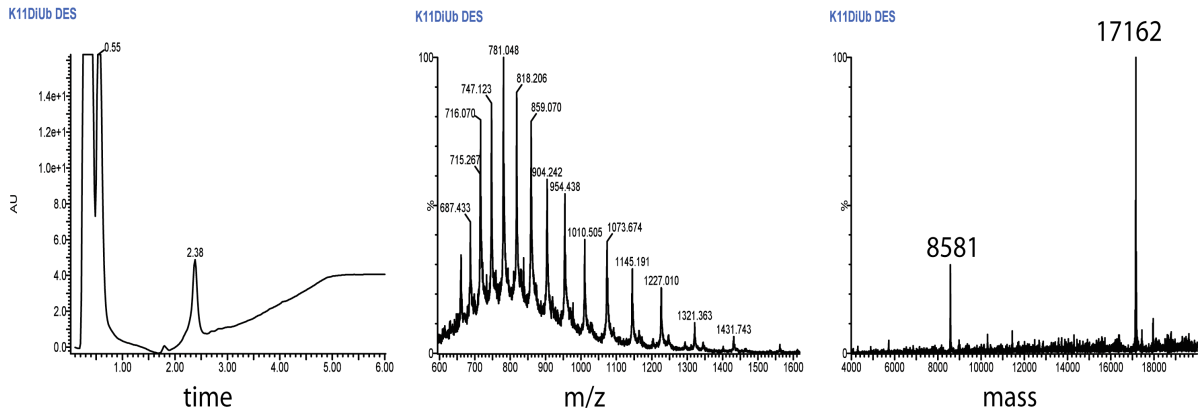

Figure S36. LC-MS analysis (program 3) of V-50 desulfurized K11-linked diUb conjugate (note that one Ub has a G76V mutation).

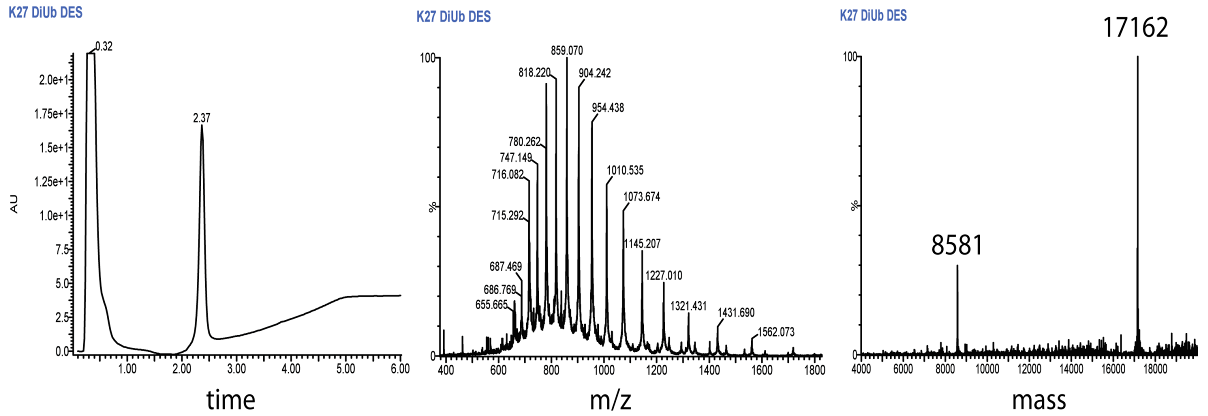

Figure S37. LC-MS analysis (program 3) of V-50 desulfurized K27-linked diUb conjugate (note that the modified Ub has a G76V mutation).

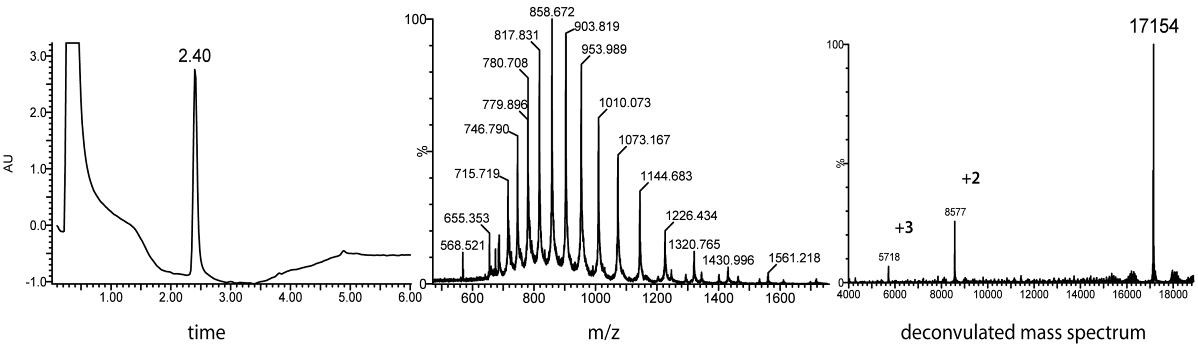

Figure S38. LC-MS analysis (program 3) of VA-044 desulfurized K29-linked diUb conjugate (note that the modified Ub has a G76V mutation).

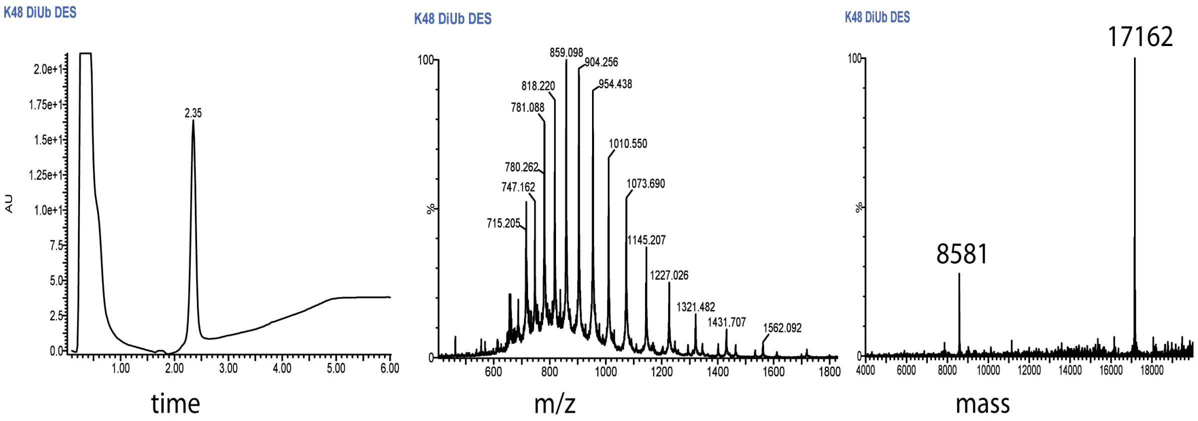

Figure S39. LC-MS analysis (program 3) of V-50 desulfurized K48-linked diUb conjugate (note that the modified Ub has a G76V mutation).

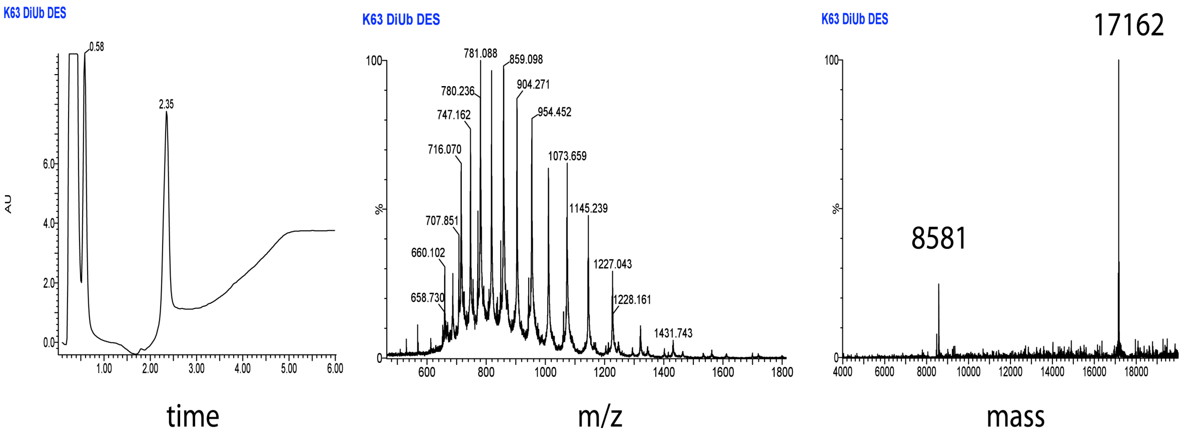

Figure S40. LC-MS analysis (program 3) of V-50 desulfurized K48-linked diUb conjugate (note that the modified Ub has a G76V mutation).

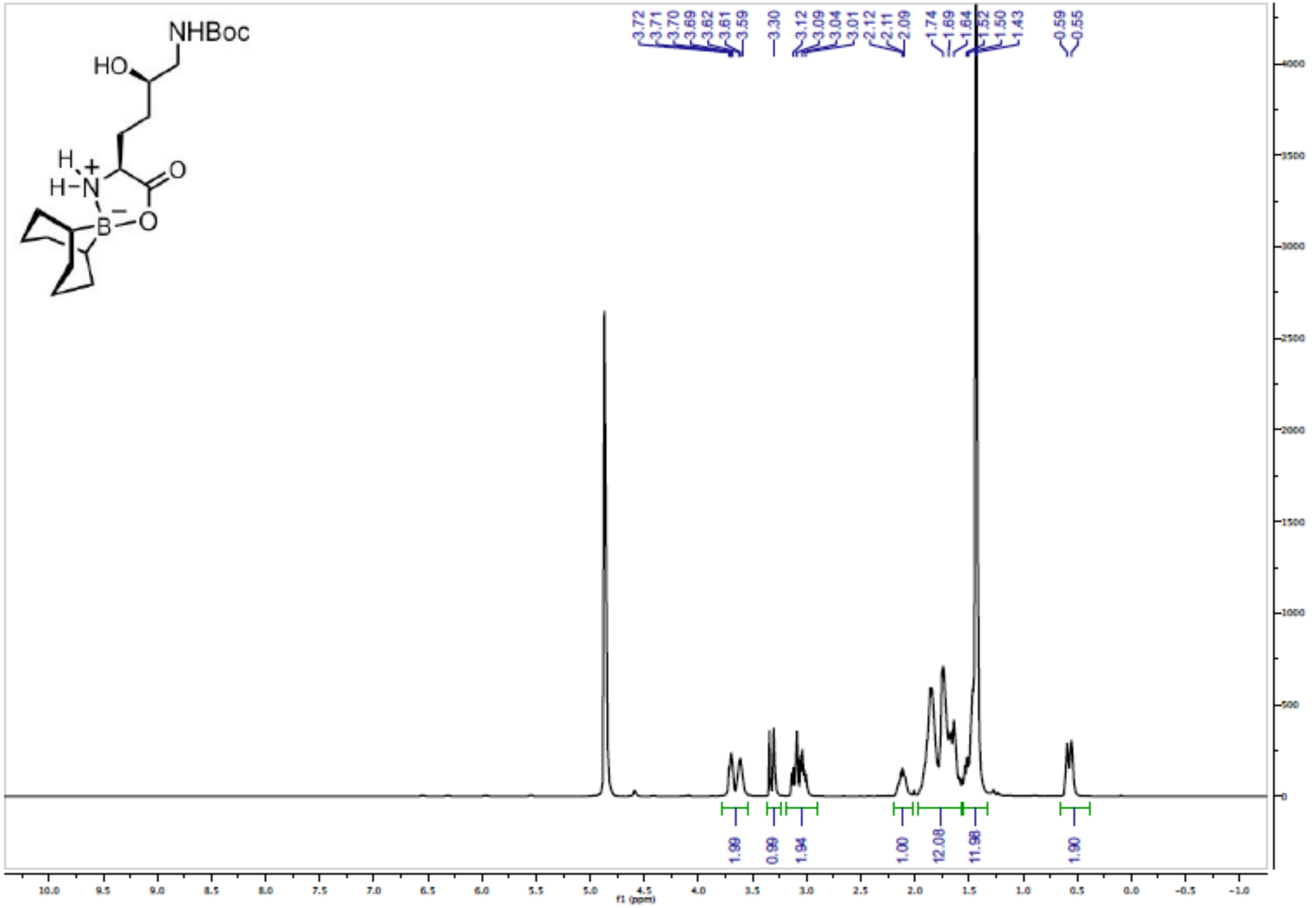
NHBoc
HO
H
H
N
B
O
O
3.72
3.71
3.70
3.69
3.62
3.61
3.59
3.30
3.12
3.09
3.04
3.01
2.12
2.11
2.09
1.74
1.69
1.64
1.52
1.50
1.43
0.59
0.55
1.99
0.99
1.94
1.00
12.08
11.98
1.90
f1 (ppm)

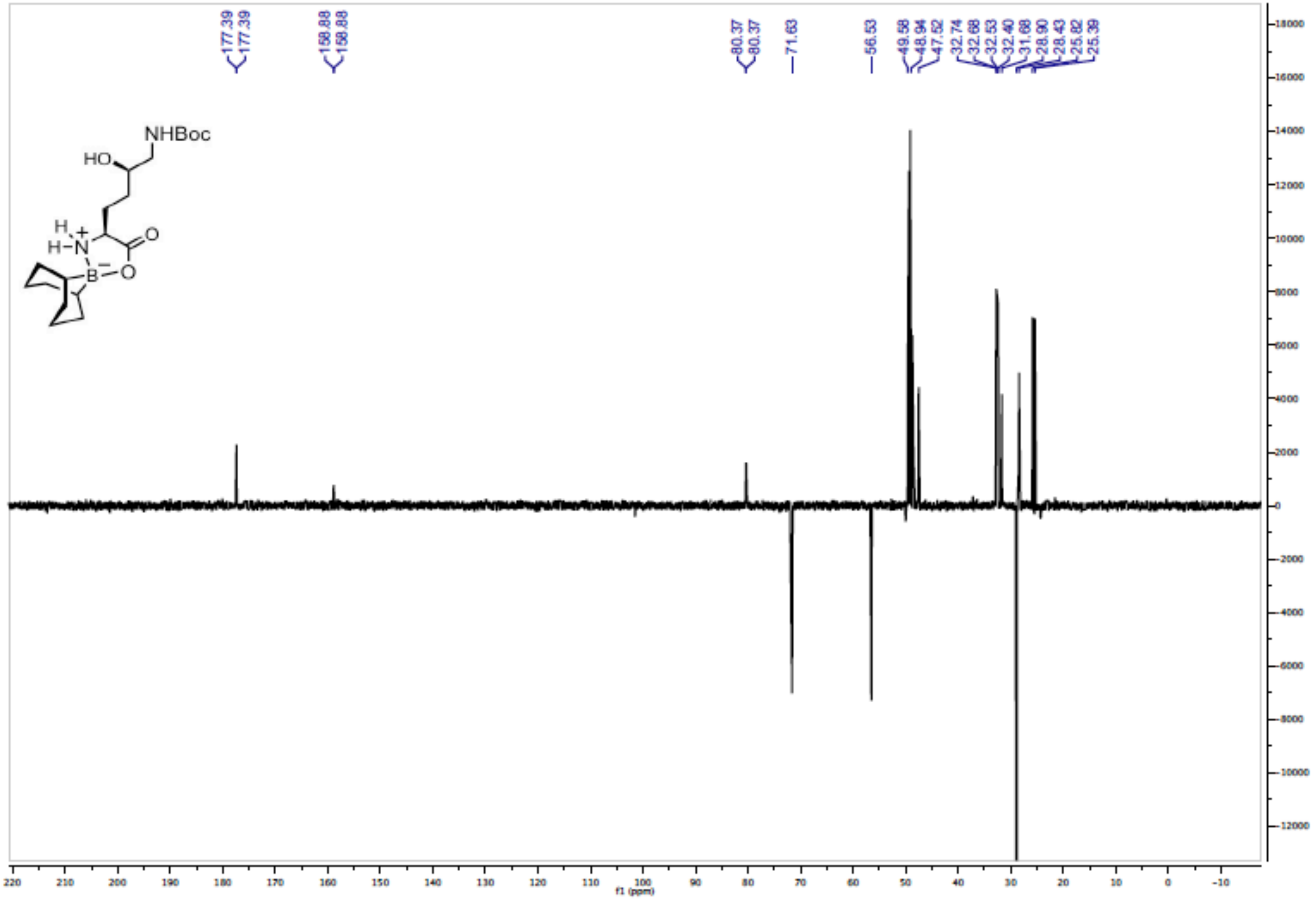
177.39
177.39
158.88
158.88
80.37
80.37
71.63
56.53
49.58
48.94
47.52
32.74
32.68
32.53
32.40
31.68
28.90
28.43
25.82
25.39
NHBoc
HO
f1 (ppm)

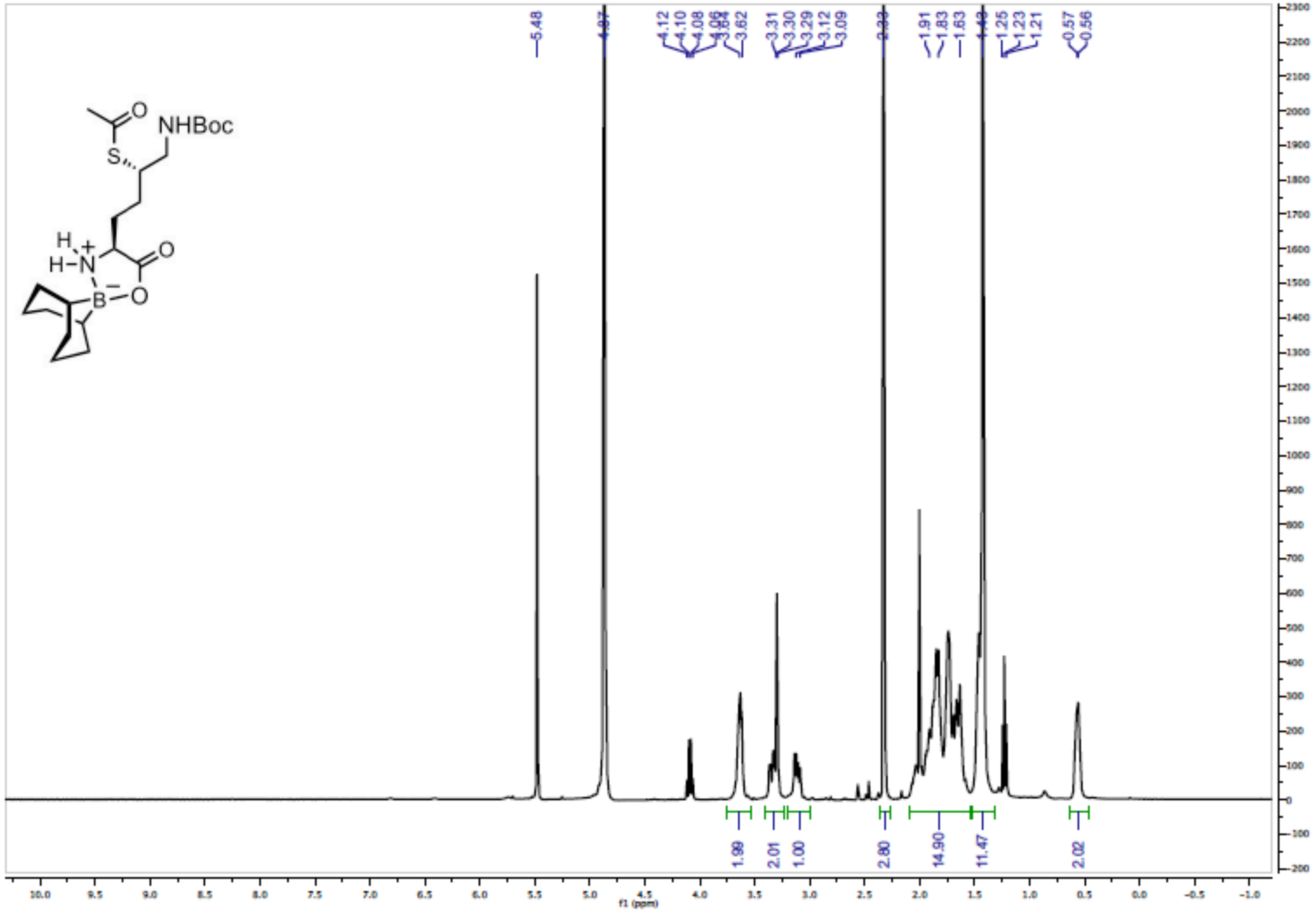
NHBoc
S
O
H
H
N
B
O
O
5.48
4.87
4.12
4.10
4.08
4.06
3.64
3.62
3.31
3.30
3.29
3.12
3.09
2.33
1.91
1.83
1.63
1.43
1.25
1.23
1.21
0.57
0.56
1.99
2.01
1.00
2.80
14.90
11.47
2.02
f1 (ppm)

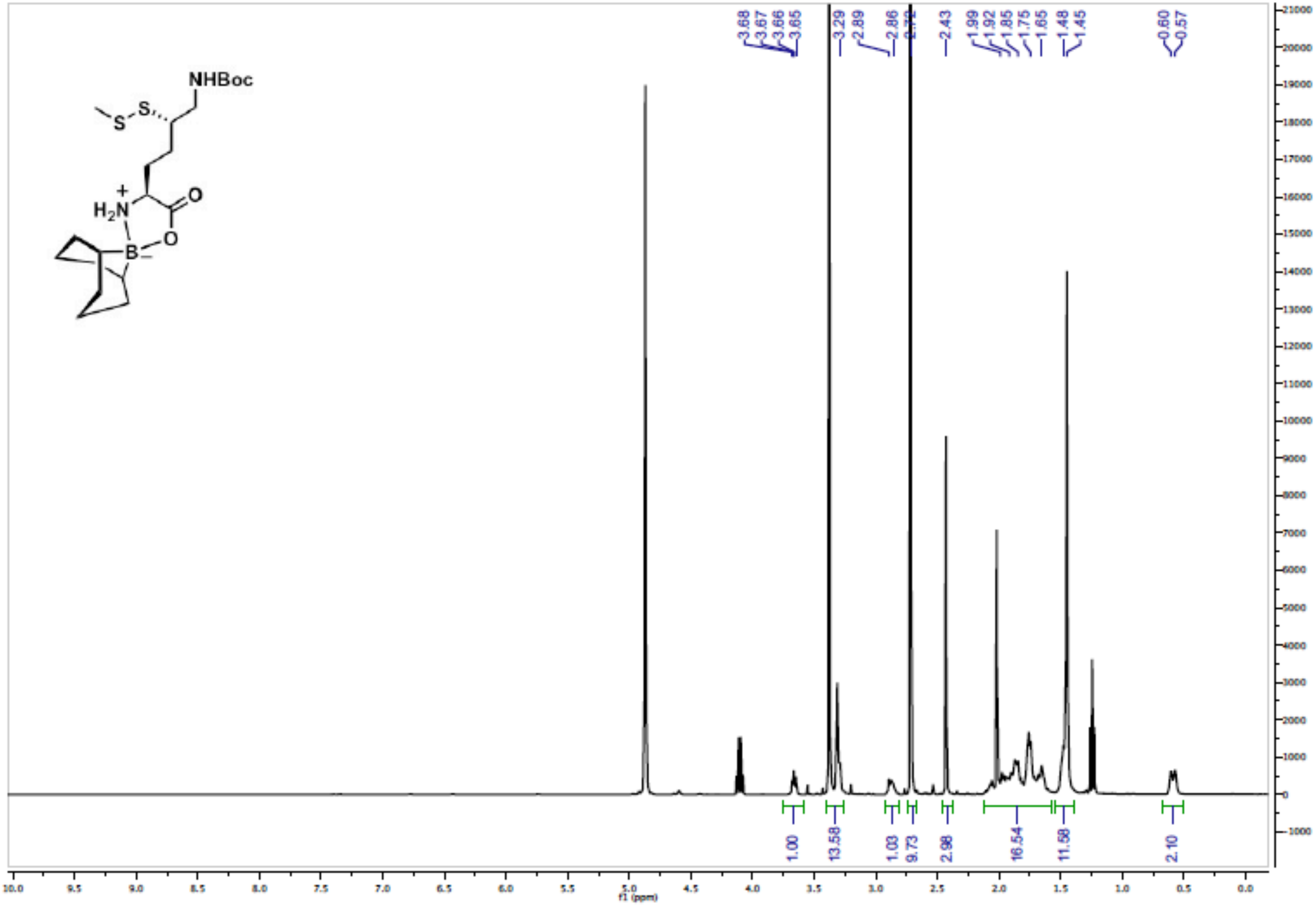
NHBoc
S
S
H_2N
O
O
B
3.68
3.67
3.66
3.65
3.29
2.89
2.86
2.72
2.43
1.99
1.92
1.85
1.75
1.65
1.48
1.45
0.60
0.57
1.00
13.58
1.03
9.73
2.98
16.54
11.58
2.10
f1 (ppm)

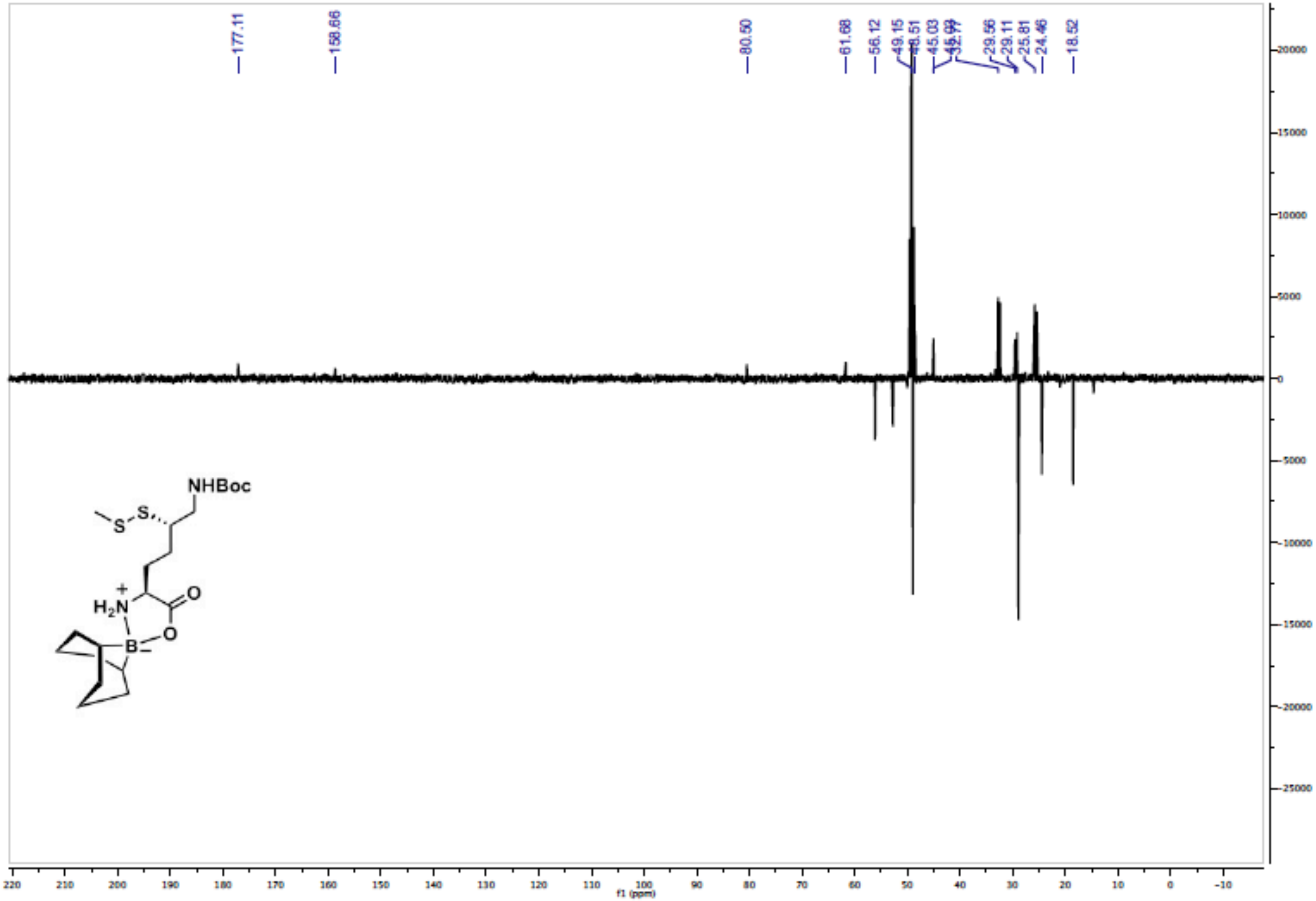
177.11
158.66
80.50
61.68
56.12
49.15
48.51
45.03
29.56
29.11
25.81
24.46
18.52
f1 (ppm)
NHBoc
S
S
H2N
O
O
B

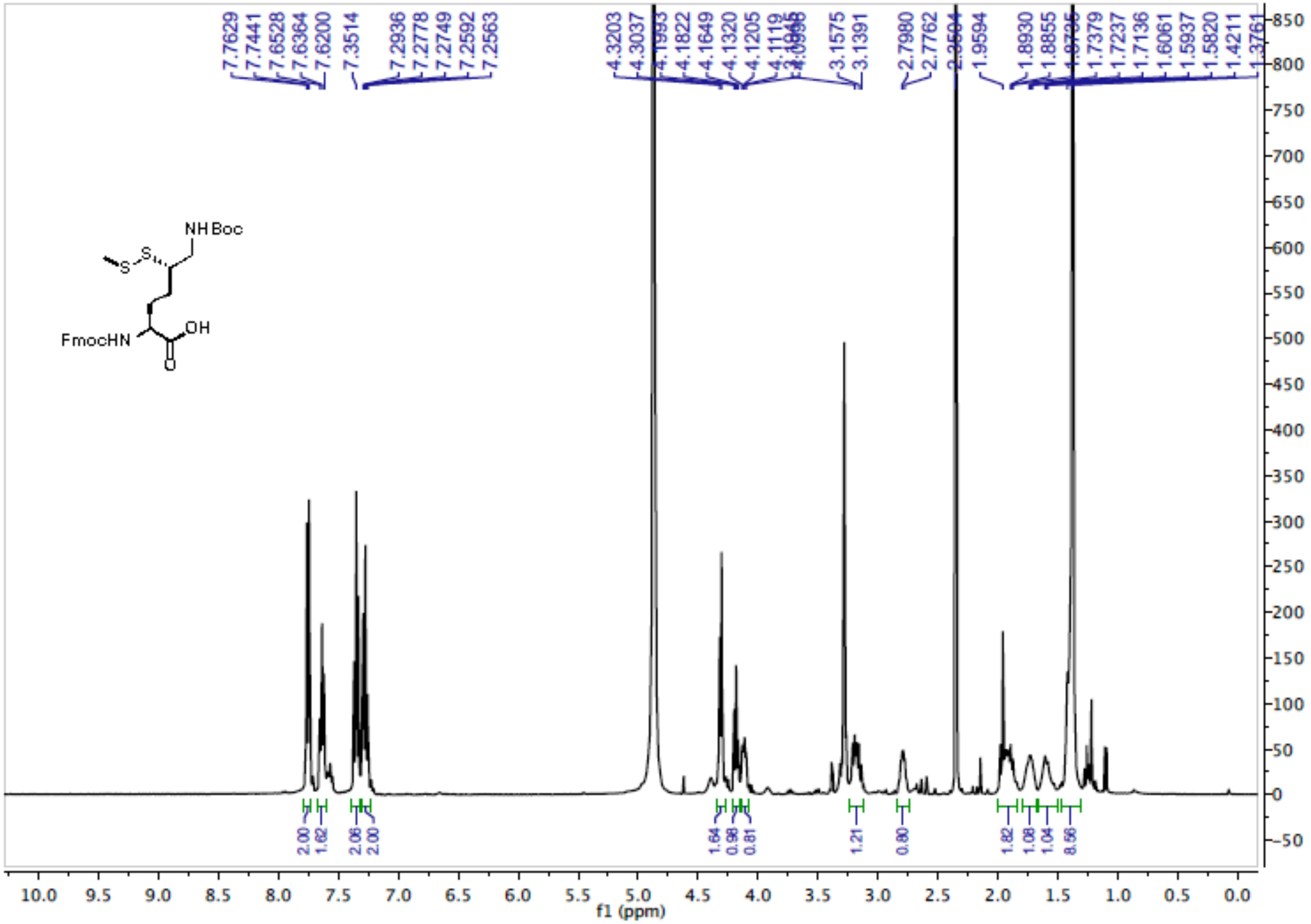
NHBoc
S
S
FmocHN
OH
O
7.7629
7.7441
7.6528
7.6364
7.6200
7.3514
7.2936
7.2778
7.2749
7.2592
7.2563
4.3203
4.3037
4.1993
4.1822
4.1649
4.1320
4.1205
4.1119
3.1575
3.1391
2.7980
2.7762
2.3504
1.9594
1.8930
1.8855
1.7379
1.7237
1.7136
1.6061
1.5937
1.5820
1.4211
1.3761
2.00
1.62
2.06
2.00
1.64
0.98
0.81
1.21
0.80
1.82
1.08
1.04
8.56
f1 (ppm)

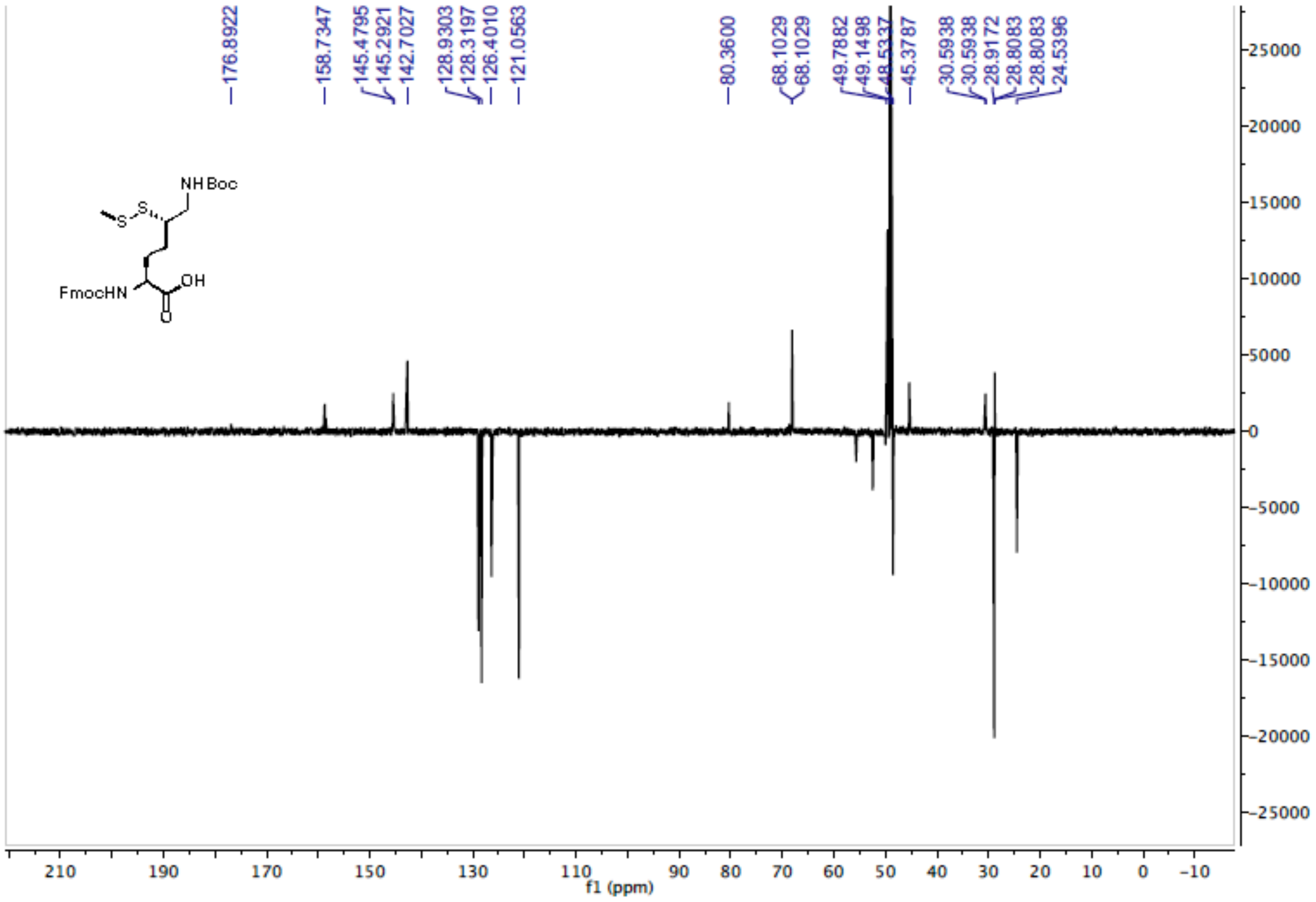
176.8922
158.7347
145.4795
145.2921
142.7027
128.9303
128.3197
126.4010
121.0563
80.3600
68.1029
68.1029
49.7882
49.1498
48.5337
45.3787
30.5938
30.5938
28.9172
28.8083
28.8083
24.5396
NHBoc
S
S
FmocHN
OH
O
f1 (ppm)
